# Effects of motor activity versus cognitive demand on asymmetries in EEG frequency bands and their relation to handedness

**DOI:** 10.3389/fnhum.2026.1796350

**Published:** 2026-07-08

**Authors:** Annakarina Mundorf, Sarah A. Merklein, Petunia Reinke, Jutta Peterburs, Stephan Getzmann, Patrick D. Gajewski, Mauro Larra, Edmund Wascher, Sebastian Ocklenburg

**Affiliations:** 1 Institute for Systems Medicine and Department of Human Medicine, MSH Medical School Hamburg, Hamburg, Germany; 2 Department of Psychology, MSH Medical School Hamburg, Hamburg, Germany; 3 Institute for Cognitive and Affective Neuroscience, MSH Medical School Hamburg, Hamburg, Germany; 4 Leibniz Research Centre for Working Environment and Human Factors (IfADo) at the Technical University of Dortmund, Dortmund, Germany; 5 German Center for Mental Health (DZPG), Partner Site Bochum/Marburg, Bochum, Germany; 6 Biopsychology, Institute for Cognitive Neuroscience, Ruhr University Bochum, Bochum, Germany

**Keywords:** alpha asymmetry, Asymmetry Index, motor preparation, oscillations, visuospatial cognition

## Abstract

**Introduction:**

Electroencephalography (EEG) is commonly used in neuroscience to study cognitive and emotional processes, often showing functional lateralization. Alpha asymmetries are frequently interpreted as a reverse marker of functional asymmetries in cognitive activation. However, the role of motor activity versus cognitive demands in driving EEG asymmetries remains unclear.

**Methods:**

To explore this, we analyzed resting-state and task-based EEG asymmetry from 610 healthy adults (ages 20–70) from the Dortmund Vital Study (ClinicalTrials.gov: NCT05155397). Participants completed three tasks with increasing hand involvement and cognitive demands: Psychomotor Vigilance, Simon, and Stroop tasks.

**Results:**

Timepoint (pre-stimulus vs. post-stimulus vs. resting-state) had the strongest impact, especially in the alpha and theta bands, with notable differences between resting-state and task-related asymmetries. Non-right-handers exhibited more dynamic shifts in asymmetry, while right-handers showed more stable lateralization patterns. Correlations between frequency bands, electrode pairs, and tasks revealed strong interhemispheric coupling in frontal and weaker coupling in central regions. Frontal post-stimulus asymmetry correlated more strongly than resting-state or pre-stimulus.

**Discussion:**

Findings suggest that EEG asymmetry is a dynamic process influenced by cognitive and/or motor activity and perceptual processes mediated by frontoparietal circuits, with implications for interpreting EEG biomarkers in both research and clinical settings. This study offers reference standards in EEG asymmetry research.

## 1 Introduction

A century ago, the psychiatrist Hans Berger achieved the first recording of spontaneous electrical activity from a human brain, marking the birth of modern electroencephalography (EEG) (Mushtaq et al., 2024). Since then, EEG has become one of the most widely utilized tools in psychology and clinical neuroscience, providing valuable insights into brain function. Early on, alpha waves were hypothesized to reflect a resting mind characterized by the absence of cognition (Mushtaq et al., 2024). Typically defined as oscillatory brain activity in the frequency range of 8–12 Hertz, alpha waves are most prominent during relaxed wakefulness with closed eyes, and commonly associated with inhibitory cortical processes (Schomer and Lopes da Silva, 2017).

Functional lateralization refers to the specialization of certain neural functions or cognitive processes predominantly in one hemisphere of the brain (Ocklenburg and Güntürkün, 2024). In the context of cognition, this implies that specific tasks, such as language processing or spatial reasoning, are primarily managed by either the left or right hemisphere, respectively (Ocklenburg et al., 2024). Moreover, EEG studies of functional lateralization have shown that greater left frontal activity is linked to increased approach motivation and reward anticipation, whereas greater right frontal activity is associated with withdrawal-related emotional states (Harmon-Jones and Gable, 2018; Reznik and Allen, 2018). Recent research has increasingly focused on understanding the underlying mechanisms driving these lateralization patterns, particularly how motor preparation and attentional processes might influence or contribute to these patterns of brain activity.

In particular, alpha asymmetries have been explored in clinical studies examining emotional valence and motivation in conditions such as depression and anxiety (Thibodeau et al., 2006; Nusslock et al., 2018; Smith et al., 2018), post-traumatic stress disorder (Meyer et al., 2018), and borderline personality disorder (for a review, see Mundorf et al., 2024a). Following Berger’s initial idea that alpha activity reflects cortical idling or an absence of active cognitive engagement, alpha asymmetries have often been interpreted as an inverse indicator of hemispheric functional specialization. In this view, greater alpha power frontally in the left hemisphere would suggest reduced cortical activation in that region, and therefore imply stronger functional activity in the right hemisphere, and vice versa (Reznik and Allen, 2018). However, this interpretation has increasingly been called into question. A growing body of research has shown that alpha oscillations are not merely passive markers of inactivity but actively contribute to key cognitive and motor processes, such as motor control, visuospatial attention, and working memory (Buzsáki and Draguhn, 2004; Klimesch et al., 2007; Morrone and Minini, 2023).

Importantly, neural oscillations across different frequency bands beyond the alpha band have been associated with distinct cognitive and affective processes. Ray and Cole (1985) proposed that alpha activity measured in the EEG reflects attentional demands during cognitive tasks, whereas beta activity rather reflects emotional and cognitive processes. Theta and delta bands, in turn, have been associated with memory encoding and deep states of cognitive processing, respectively (Addante et al., 2011; Züst et al., 2019). Additionally, alpha peak frequency adaptation, the dynamic adjustment of the brain’s dominant alpha frequency in response to cognitive demands, may influence perception, facilitate memory access, and suppress irrelevant sensory inputs during attention tasks (Mierau et al., 2017).

While much of the research has focused on the absolute power and functional role of these oscillations, asymmetries in their distribution between hemispheres have also received growing attention. For example, several studies have highlighted the role of alpha and theta asymmetries in cognitive and memory performance (Katerman et al., 2022; Pavlov and Kotchoubey, 2022; Tan et al., 2024). Notably, alpha band activity can exhibit both increases and decreases in relative power, whereas changes in other EEG bands are generally task- and region-dependent. These bands often show increases in response to stimuli or cognitive demands, although decreases have also been reported depending on the oscillatory band and cortical localization (Klimesch, 1999, 2012; Harmony, 2013; Katerman et al., 2022; Codispoti et al., 2023; Tan et al., 2024; Ünsal et al., 2024). Furthermore, research by Ocklenburg et al. (2019) demonstrated strong positive correlations between alpha, beta, theta, and delta frequency bands. Despite these advances, it remains unclear whether observed asymmetries primarily reflect domain-specific functional processes or are influenced by concurrent motor and attentional demands.

Therefore, the neurophysiological role of alpha asymmetry remains unclear, raising questions about whether it reflects domain-specific functional asymmetries, underlying motor activity, or other processes. In particular, the extent to which these oscillatory patterns reflect true functional asymmetries versus being influenced by motor activity remains unclear, limiting their clinical interpretability. Resolving this ambiguity is critical for establishing alpha asymmetry and related oscillatory patterns as reliable indicators of emotional or cognitive states in diagnostic and therapeutic contexts.

Recent studies have highlighted the distinct roles of the left and right hemispheres in cognitive processing, with the right hemisphere playing a key role in prioritizing information and the left hemisphere specializing in decision-making and perceptual model-building (Bartolomeo et al., 2024). In particular, right-hemispheric fronto-parietal networks have been strongly implicated in spatial attention and attentional prioritization, forming a key component of right-lateralized attention systems (Corbetta and Shulman, 2002). Furthermore, frontal midline theta activity has been linked to cognitive control, performance monitoring, and working memory demands, typically involving medial prefrontal regions (Cavanagh and Frank, 2014). Beta-band activity in sensorimotor regions typically decreases during movement and increases again after movement ends. This post-movement increase has been interpreted to reflect a temporary suppression of motor activity and a reset of the motor system after action execution (Neuper and Pfurtscheller, 2001). These hemispheric divisions are supported by clinical observations, particularly in neglect syndrome, where disruptions in attentional processing align with hemispheric dominance (Doricchi and Tomaiuolo, 2003; Thiebaut de Schotten et al., 2014; Lunven et al., 2015).

Moreover, individual factors such as handedness and age can significantly influence lateralization patterns, making them essential variables to consider in EEG research. Ocklenburg et al. (2019) demonstrated that right-handed individuals exhibit stronger rightward alpha asymmetry, reflecting greater left hemisphere activity, whereas non-right-handed individuals show more dynamic shifts in asymmetry across multiple frequency bands, such as theta, alpha, and beta (Ocklenburg et al., 2019).

Hemispheric lateralization is also not fixed across the lifespan. Learmonth et al. (2017) found that younger adults typically exhibit a leftward visuospatial bias, driven by right-hemispheric dominance, but this bias shifts toward the right with age, reflecting a decline in the right hemisphere’s role in spatial attention (Learmonth et al., 2017). This shift is reflected in EEG findings, in which older adults show reduced right-hemispheric specialization, leading to more bilateral processing. These age-related changes in lateralization may be indicative of compensatory mechanisms or early neurodegenerative changes, aligning with the neural dedifferentiation hypothesis of aging (Goh, 2011; Koen and Rugg, 2019). These findings highlight the importance of considering age, handedness, and other individual factors when investigating lateralization in EEG research, as they significantly influence neural organization and cognitive performance. Understanding how these factors interact can provide more accurate and reliable insights into hemispheric asymmetries, especially in clinical settings.

The present study aimed to address a crucial gap in our understanding of EEG asymmetry by investigating whether functional lateralization in EEG signals across multiple frequency bands is primarily driven by task-specific processes or influenced by motor and cognitive activity. The main aim of the present work, therefore, was to explore how alpha, beta, delta, and theta band asymmetries vary across tasks with differing motor and cognitive demands in a large sample of healthy adults. The objective behind this was to get a better understanding of which factors influence task-based oscillation asymmetries in the EEG signal.

Considering that reduced lateralization for spatial attention has been observed in older populations (Learmonth et al., 2017), and that potential sex differences in hemispheric asymmetry are discussed in the literature (Hirnstein et al., 2019), we also explored how age, sex, and handedness affect functional lateralization. The main contributions of the present study are (i) a systematic comparison of task-based asymmetry across multiple frequency bands, (ii) the dissociation of motor preparation and execution, and (iii) the evaluation of interindividual factors such as age, sex, and handedness in a large population-based sample.

## 2 Methods

### 2.1 Participants

All data were taken from the Dortmund Vital Study, a population-based, longitudinal study that focuses on healthy working adults (Clinicaltrials.gov NCT05155397) (Gajewski et al., 2022; Mundorf et al., 2023). The sample consists of healthy participants ranging from 20 to 70 years of age, with no restrictions in terms of education or occupation (Gajewski et al., 2022). Data from 610 participants (232 men, 378 women) with a mean age of 43.83 years were available for analysis. The Dortmund Vital Study received ethical approval in October 2015 from the local Ethics Committee of the Leibniz Research Centre for Working Environment and Human Factors, Dortmund (IfADo) and conforms to the Code of Ethics of the World Medical Association (Declaration of Helsinki). The participants gave their written informed consent before any study protocol was commenced. Of note, data availability varied across tasks and frequency bands, so the effective sample sizes differed between analyses. For each analysis, only participants with valid data for the respective measures were included. Corresponding sample sizes are reported in the respective results tables.

### 2.2 Handedness assessment and classification

Handedness was assessed with the Edinburgh Handedness Inventory (Oldfield, 1971), and for each participant, a lateralization quotient (LQ) was calculated with the formula: LQ = ((R − L)/(R + L)) × 100, with R reflecting the number of right-hand preferences and L measuring the number of left-hand preferences as described by Oldfield (1971). Based on the LQ, three handedness groups were formed: left-handedness: LQ = −100 to −61, mixed-handedness: LQ = −60 to +59, and right-handedness: LQ = 60 to 100 (Mundorf et al., 2024b). This grouping resulted in 29 left-handers, 96 mixed-handers, and 482 right-handers.

### 2.3 EEG tasks description

In this study, we analyzed EEG data from three cognitive tasks that differ systematically in both motor requirements and cognitive load: the Psychomotor Vigilance Task (PVT), the Simon Task, and the Stroop Task (for detailed descriptions, see below). The PVT, which involves simple reaction-time responses with the dominant hand and the index finger, places minimal cognitive demands and primarily measures sustained attention. In contrast, the Simon Task introduces moderate cognitive load by requiring spatial conflict resolution and bimanual choice responses based on stimulus identity rather than location. The Stroop Task imposes the highest cognitive load, necessitating interference control and semantic conflict resolution, with participants selecting one of four color-based responses using both hands and four fingers. This gradation in cognitive complexity across tasks provides a robust framework for examining the interactions of cognitive and motor processes in EEG data.

#### 2.3.1 Psychomotor vigilance task

The classical PVT is a simple reaction time paradigm in which participants respond as quickly as possible to a visual stimulus presented at random interstimulus intervals, typically without additional task demands (Dinges and Powell, 1985). The PVT is commonly used to measure sustained or vigilant attention by recording response times to visual stimuli that occur at random interstimulus intervals (2, 3, 5, and 8 s) over 10 min with a total of 132 trials (32 for each interval) (Dinges and Powell, 1985; Wascher et al., 2022). To increase the sensitivity of the test to more subtle changes (as they may occur in a healthy population), a modified version of the PVT was used, in which as stimulus, a white disk (80 cd/m^2^), was presented on a dark grey background (20 cd/m^2^) in the center of the screen (visual angle: 3°) for 150 ms. Participants were asked to respond as fast as possible via button press with the dominant hand to any stimulus appearing on the screen (Wascher et al., 2022). The task required a simple response using one response button.

#### 2.3.2 Simon task

The Simon task allows the examination of the contribution of conflict adaptation and feature integration effects on sequential modulations of the Simon effect (i.e., the difference in accuracy or response time between trials where the stimulus and response occur on the same side versus opposite sides), by using different spatial dimensions (Wascher and Wolber, 2004; Hoppe et al., 2017; Sabo et al., 2026). Here, stimuli were presented in both the vertical and the horizontal dimension with vertically positioned response keys (upper and lower response keys). Response keys were pressed with the left or right hand, respectively. During trials, one of two possible target stimuli (letter S or X) appeared either on the horizontal axis (left or right from the center) or on the vertical axis (above or below the center). Stimuli were previously mapped to response keys (upper or lower) irrespective of their spatial position during trials (Hoppe et al., 2017). This task required a two-choice response using two response buttons.

#### 2.3.3 Stroop task

Susceptibility to interference was examined in the color-word interference Stroop test. In this test, the words “red,” “green,” “yellow,” and “blue” (5–7 mm wide x 10 mm high) were displayed as stimuli on a black computer screen in one of the four colors. In 50% of the trials, the color of the displayed word matched its meaning (congruent), while in the other half, the word’s color and meaning were different (incongruent). To respond to each target stimulus, participants needed to press one of four designated keys on a response box using the index and middle fingers of both hands (Gajewski et al., 2020; Larra et al., 2024; Sabo et al., 2026).

### 2.4 Recording and EEG data processing

The EEG experiments of the Dortmund Vital Study took place on two separate days (for details see Gajewski et al., 2022). Two of the tasks considered here were performed on day 1 of testing (PVT and Simon task), and one task (Stroop task) on day 2. EEG recordings on day 1 were made using a 64-channel actiCap system (Brain Products GmbH, Munich, Germany) with a sampling rate of 1,000 Hz, and FCz as the online reference electrode. EEG recordings on day 2 were made using a 32-channel BioSemi system (BioSemi B. V., Amsterdam, The Netherlands) with a sampling rate of 2048 Hz. Here, a common mode sense (CMS) active electrode and a driven right leg (DRL) passive electrode are employed. Together, these two electrodes create a feedback loop that regulates the subject’s average potential. The reference and ground electrodes are incorporated into the CMS and DRL loop (for details, see Metzen et al., 2022; Kleinert et al., 2024; Larra et al., 2024; Sabo et al., 2026). The use of two EEG systems was driven by technical constraints related to the evolution of the Dortmund Vital study, requiring different recording setups across study phases (Gajewski et al., 2022).

The same participants were tested on both days, with sessions scheduled between 9.30 and 10.00 a.m. on each day. Participants were seated comfortably in an electrically shielded, sound-attenuated room. Electrode positions conformed to the international 10–20 System, and impedances were kept below 10 kΩ. Electrolytic gel was applied to each electrode side to maintain low impedance. In addition to the EEG recorded during the cognitive tasks, resting-state EEG was recorded on day 1 during a rest period before and after the block of cognitive tasks, including 2 min of eyes-open and 2 min of eyes-closed (Getzmann et al., 2024).

EEG data were processed in Matlab 2018b using EEGLab (Delorme and Makeig, 2004). Signals were band-pass filtered using a zero-phase FIR filter (resting-state: 1–30 Hz; tasks: 0.1–40 Hz) and re-referenced to a common reference electrode. Bad channels were identified using automated procedures based on a robust average reference, implemented via the PrepPipeline (Bigdely-Shamlo et al., 2015) and defined by criteria such as abnormal signal deviation, reduced correlation with neighboring electrodes, and elevated noise levels, and subsequently interpolated. After segmenting the continuous data into 2-s epochs (−1,000 to 1,000 ms relative to stimulus onset), corrupted epochs were automatically rejected using the EEGLAB function pop_autorej with a voltage threshold of ±500 μV; with on average, 94.1% (SD = 4.6) of epochs retained for EEG system 1 and 96.1% (SD = 4.1) for EEG system 2 for resting-state data (Metzen et al., 2022). Baseline correction was applied using a pre-stimulus interval. Ocular artifacts were removed using independent component analysis. ICA was performed on the continuous data, and components representing eye-related activity were identified using ICLabel (Pion-Tonachini et al., 2019) and excluded if classified as eye components with a probability >80%.

**Figure 1:**
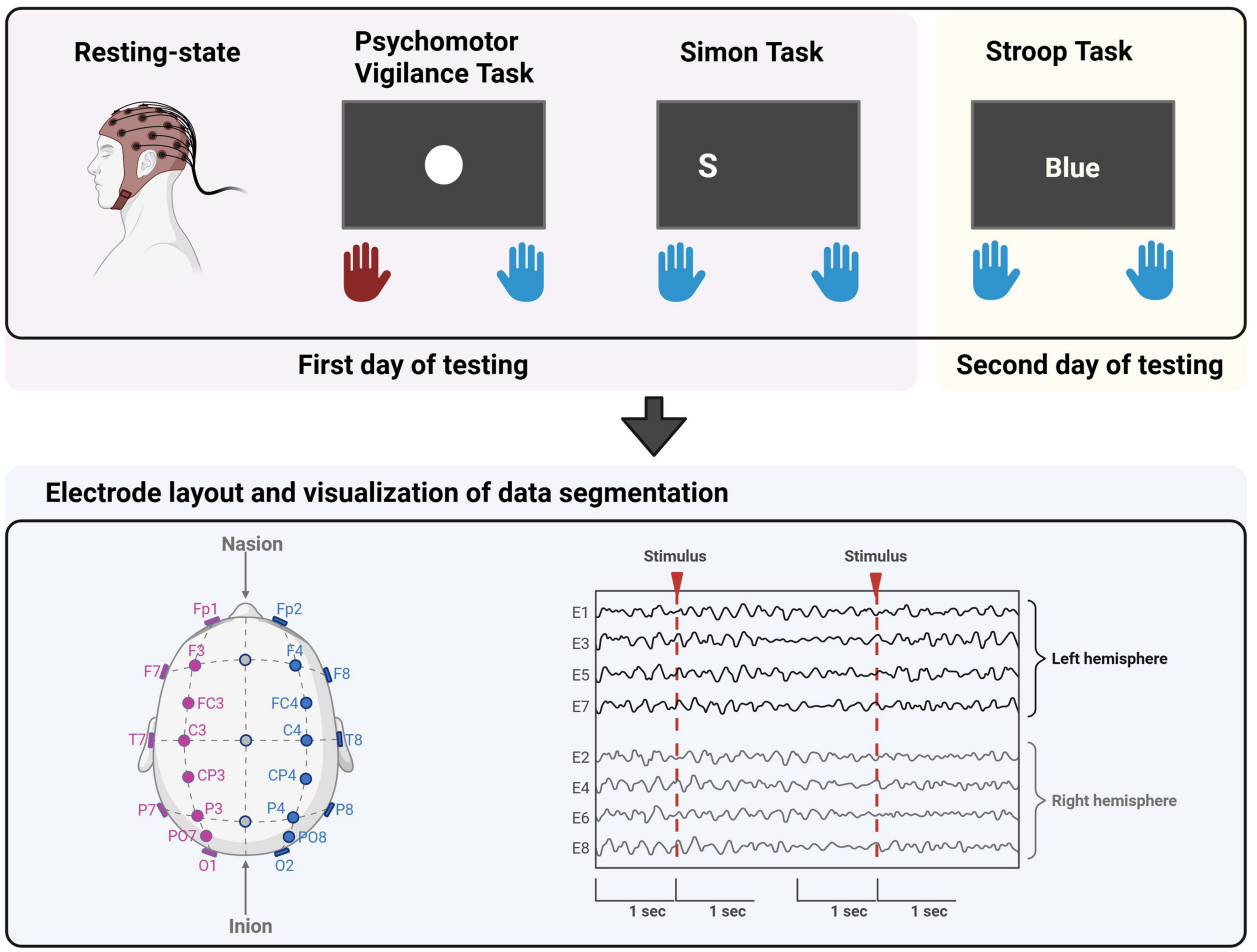
Schematic overview of the experimental procedure, cognitive tasks, and EEG recording windows. The upper panel illustrates the sequence of tasks across two testing days. On Day 1, resting-state EEG was recorded first (with eyes open and closed), followed by EEG during the Psychomotor Vigilance Task and the Simon Task. On Day 2, participants performed the Stroop Task. Each task is visualized based on its stimulus display, and required hand responses are depicted. The Psychomotor Vigilance Task involved a simple button press with the dominant hand only (indicated in red), while the Simon and Stroop Tasks required bimanual responses using both hands, reflecting increasing cognitive and motor demands across tasks. The lower panel depicts the electrode layout with eleven electrode pairs. Electrodes on the left side of the head are colored pink, and electrodes on the right side are colored light blue. Also, the segmentation windows for task-related recordings are visualized. EEG was segmented into 1 s before (pre-stimulus) and 1 s after (post-stimulus) stimulus onset. These time windows allowed for the assessment of neural asymmetry related to motor preparation (pre-stimulus) and motor/cognitive execution (post-stimulus). Created in BioRender. Mundorf et al. (2025) https://BioRender.com/530odl5.

For the present study, EEG was analyzed at 22 electrodes that form 11 electrode pairs as follows: frontal electrode pairs F3/F4, F7/F8, Fp1/Fp2, central electrode pairs T7/T8, FC3/FC4, C3/C4, CP3/CP4, and parieto-occipital electrode pairs P3/P4, P7/P8, PO3/PO4, O1/O2. Two time periods were analyzed for each task, that is, 1 s before (pre-stimulus) and 1 s after stimulus presentation (post-stimulus). Thereby, the effects of motor preparation and execution on frequency band asymmetry were differentiated. In addition, resting-state EEG data from day 1 were analyzed, and resting-state recordings from multiple timepoints were combined to obtain a more reliable estimate (Hagemann et al., 2002). This approach was in line with the finding that EEG alpha asymmetries are less affected by a cognitive task than EEG alpha power, and that EEG alpha asymmetries before and after a task show acceptable test–retest reliability (Mathewson et al., 2015; Kleinert et al., 2024). Figure 1 provides a schematic overview of the experimental tasks, EEG recording timepoints, bilateral electrode placement used for asymmetry calculations, and the 1-s pre- and post-stimulus analysis windows.

### 2.5 EEG Asymmetry Index calculation

EEG asymmetry indices (AI) of frequency power were calculated for each electrode pair (F3/F4, F7/F8, Fp1/Fp2, T7/T8, FC3/FC4, C3/C4, CP3/CP4, P3/P4, P7/P8, PO3/PO4, O1/O2), frequency band (alpha: 8–12 Hz, beta: 12-30 Hz, theta: 4-8 Hz, delta: 0.5-4 Hz), task (PVT, Simon, Stroop), and timepoint (resting-state, pre- and post-stimulus) separately using the formula: AI = (ln[right electrode] – ln[left electrode]). Thus, positive scores reflect higher right-hemispheric power, and negative scores reflect higher left-hemispheric power. While different definitions exist for the range of the delta band, 0.5 to 4 Hz is a common choice in the literature (Swarnkar et al., 2006; Spironelli and Angrilli, 2009; Attar, 2022).

### 2.6 Statistical analyses

For each task and frequency band, an ANOVA was performed with the dependent variable AI, and the independent variables electrode pair (F3/F4, F7/F8, Fp1/Fp2, T7/T8, FC3/FC4, C3/C4, CP3/CP4, P3/P4, P7/P8, PO3/PO4, O1/O2), timepoint (pre-stimulus, post-stimulus, resting-state), centered age as covariate, and the between subject variable sex. Separate analyses were performed for right-handers (LQ = 60 to 100) and for non-right-handers to account for possible effects of handedness on EEG asymmetry (see 2.1 Participants description). For the group of non-right-handers, left- and mixed-handers (LQ < 60 to −100) were included, given that both the left- and mixed-handed groups were too small to run subgroup analyses. This grouping decision was motivated both by statistical power considerations due to limited sample sizes in the left- and mixed-handed subgroups and by the conceptual and clinical relevance of distinguishing between right- and non-right-handed individuals, as suggested by previous meta-analytic evidence (e.g., Nastou et al., 2022; Ocklenburg et al., 2024; Mundorf et al., 2025; Packheiser et al., 2025). Following the ANOVAs, Bonferroni-corrected *post hoc t*-tests were performed to further investigate the found effects. In a separate step, Pearson’s correlations were computed separately for each task, pre- and post-stimulus, and resting-state data with the calculated AI between frequency bands for each electrode pair. All statistical analyses were performed in R Studio (RStudio Team, 2020; Version 2024.12.0).

The interpretation of results focused on the reported effect sizes, as suggested by Tomczak and Tomczak (2014). Accordingly, effect sizes derived from the ANOVA are presented as partial eta squared (*η*_p_^2^) and classified according to Cohen (1988) as negligible (*η*_p_^2^ < 0.01), small (0.01 ≤ *η*_p_^2^ < 0.06), medium (0.06 ≤ *η*_p_^2^ < 0.14), or large (*η*_p_^2^ ≥ 0.14). For Pearson’s *r*, effect sizes are defined as small (0.1 ≤ *r* < 0.3), medium (0.3 ≤ *r* < 0.5), or large (*r* ≥ 0.5); for Cohen’s *d*, effect sizes were characterized as small (±0.2 ≤ *d* < ±0.5), medium (±0.5 ≤ *d* < ±0.8), or large (*d* ≤ ±0.8) (Cohen, 1988). However, *p*-values were reported for completeness and to ensure comparability with other studies.

## 3 Results

### 3.1 Analyses including only right-handers

#### 3.1.1 PVT

The ANOVA results revealed significant main effects of age, electrode pair, and timepoint on AI, along with significant interactions between age and electrode pair, and electrode pair and timepoint. These factors significantly influence EEG asymmetry across the frequency bands. Only effects with at least medium effect sizes are described in the text; complete statistical details are provided in Table 1; *post-hoc t*-test analyses can be found in Supplementary material 1, and separate ANOVAs for electrode pairs in Supplementary material 2. Heatmaps of effect sizes (*η*_p_^2^) derived from the ANOVAs on electrode pair and timepoint per task across the different frequency bands are presented in Supplementary material 3 in Figure S1, sex-specific means in Supplementary material 4, and age-related visualizations of AI per frequency band in Supplementary material 3 in Figures S7–S10.

**Table 1:** ANOVA results for the PVT in right-handed participants across frequency bands.

Parameter	*df*	*F*	*η*_p_^2^	*p*-value
Alpha (*N* = 428)				
Sex	1	0.001	<0.001	0.974
Age	1	13.486	0.037	<0.001***
Electrode pair	5.195	43.076	0.110	<0.001***
Sex * Electrode pair	5.195	1.257	0.004	0.278919
Age * Electrode pair	5.195	6.770	0.019	<0.001***
Timepoint	1.750	22.543	0.061	<0.001***
Sex * Timepoint	1.750	1.692	0.005	0.189
Age * Timepoint	1.750	13.061	0.036	<0.001***
Electrode pair * Timepoint	7.785	24.345	0.065	<0.001***
Sex * Electrode pair * Timepoint	7.785	1.534	0.004	0.143
Age * Electrode pair * Timepoint	7.785	3.756	0.011	<0.001***
Beta (*N* = 417)				
Sex	1	6.251	0.018	0.013*
Age	1	1.449	0.004	0.230
Electrode pair	5.869	2.131	0.006	0.049*
Sex * Electrode pair	5.869	1.411	0.004	0.208
Age * Electrode pair	5.869	3.763	0.011	0.001***
Timepoint	1.298	33.215	0.089	<0.001***
Sex * Timepoint	1.298	6.002	0.017	0.009*
Age * Timepoint	1.298	0.116	<0.001	0.799
Electrode pair * Timepoint	6.327	9.950	0.029	<0.001***
Sex * Electrode pair * Timepoint	6.327	0.709	0.002	0.650
Age * Electrode pair * Timepoint	6.327	1.873	0.006	0.078
Delta (*N* = 372)				
Sex	1	3.205	0.011	0.074
Age	1	4.162	0.014	0.042
Electrode pair	6.586	9.392	0.031	<0.001***
Sex * Electrode pair	6.586	1.427	0.005	0.194
Age * Electrode pair	6.586	1.458	0.005	0.182
Timepoint	1.332	22.589	0.071	<0.001***
Sex * Timepoint	1.332	1.129	0.004	0.306
Age * Timepoint	1.332	0.474	0.002	0.546
Electrode pair * Timepoint	8.026	5.696	0.019	<0.001***
Sex * Electrode pair * Timepoint	8.026	1.028	0.004	0.412
Age * Electrode pair * Timepoint	8.026	1.067	0.004	0.383
Theta (*N* = 416)				
Sex	1	0.111	<0.001	0.739
Age	1	13.439	0.038	<0.001***
Electrode pair	5.373	7.713	0.022	<0.001***
Sex * Electrode pair	5.373	1.244	0.004	0.284
Age * Electrode pair	5.373	3.534	0.010	0.003**
Timepoint	1.705	62.016	0.155	<0.001***
Sex * Timepoint	1.705	1.011	0.003	0.355
Age * Timepoint	1.705	2.017	0.006	0.141
Electrode pair * Timepoint	6.819	5.048	0.015	<0.001***
Sex * Electrode pair * Timepoint	6.819	1.313	0.004	0.241
Age * Electrode pair * Timepoint	6.819	1.600	0.005	0.133

*p < 0.05 (uncorrected), **p < 0.01, ***p < 0.001. The reported statistics include degrees of freedom (*df*), *F*-values (*F*), and *p*-values (*p*). Effect sizes are given as partial eta2 (*η*_p_^2^) and characterized according to Cohen (1988) as negligible: *η*_p_^2^ < 0.01, small: 0.01 ≤ *η*_p_^2^ < 0.06, medium: 0.06 ≤ *η*_p_^2^ < 0.14 (light green), or large: *η*_p_^2^ ≥ 0.14 (dark green). The sample size (*N*) is given in the table for each frequency band.

In the alpha frequency band, significant main effects on AI were observed for electrode pair placement (*η*_p_^2^ = 0.110) and timepoint (*η*_p_^2^ = 0.061). In addition, a significant interaction between electrode pair and timepoint emerged (*η*_p_^2^ = 0.065). *Post-hoc* analyses comparing resting, pre- and post-stimulus AIs separately for each electrode pair revealed that the AI was negative and greater in magnitude pre-stimulus and post-stimulus for P7/P8 and T7/T8, indicating stronger left-hemispheric asymmetries at these timepoints. For P3/P4, the AI was less negative pre-stimulus, but more negative post-stimulus, suggesting a shift toward stronger left-hemispheric asymmetry following stimulus presentation. Similarly, at PO3/PO4 and CP3/CP4, the AI was negative post-stimulus, indicating stronger left-hemispheric asymmetries compared to the pre-stimulus period. For F7/F8, the AI was positive and larger post-stimulus compared to pre-stimulus, indicating stronger right-hemispheric asymmetry after the task. Additionally, significant interactions were found between age and timepoint (*η*_p_^2^ = 0.036), indicating that age-related changes in asymmetry varied across task conditions. In resting-state, older participants exhibited a trend toward increased right-hemispheric alpha power, as reflected by a positive association between age and AI across several electrode pairs. This age-related change was less pronounced during the pre-stimulus period, with regression slopes near zero, suggesting minimal asymmetry changes with age. Following task completion, the age-related increase in rightward asymmetry partially re-emerged, particularly over fronto-temporal and central electrode pairs (e.g., T7/T8, FC5/FC6). These findings suggest that the relationship between age and alpha asymmetry is dynamic and modulated by cognitive state or task engagement (see Supplementary material 3 in Figure S7).

In the beta frequency band, timepoint showed a significant main effect (*η*_p_^2^ = 0.089), while all other effects remained below the threshold for medium effect sizes. *Post-hoc* comparisons indicated significantly higher AI values post-stimulus compared to resting-state across several electrode pairs, including FC3/FC4, F7/F8, F3/F4, and P7/P8. Additionally, both FC3/FC4 and F7/F8 exhibited a significant increase in AI from pre- to post-stimulus, suggesting a sustained asymmetry effect following task performance. Conversely, electrode pairs such as P3/P4 and PO3/PO4 showed significantly lower AI post- compared to pre-stimulus, indicating reduced asymmetry following task completion. The most pronounced timepoint-related AI increase from pre- to post-stimulus was observed at FC3/FC4, highlighting enhanced right-hemispheric engagement during post-stimulus recovery.

In the delta frequency band, only timepoint exhibited a significant main effect (*η*_p_^2^ = 0.071), while all other effects fell below the medium effect size threshold. *Post-hoc* comparisons revealed significantly higher AI post-stimulus compared to resting-state across multiple electrode pairs, including FC3/FC4, F7/F8, P7/P8, C3/C4, CP3/CP4, Fp1/Fp2, and F3/F4. The largest timepoint-related AI increase from resting-state to post-stimulus was observed at FC3/FC4, indicating a shift toward right-hemispheric dominance.

Similarly, in the theta frequency band, a significant main effect of timepoint was observed (*η*_p_^2^ = 0.155). *Post-hoc* analyses comparing AI of resting, pre- and post-stimulus separately for each electrode pair showed significantly higher AI post-stimulus compared to resting-state across multiple electrode pairs, including T7/T8, F7/F8, P7/P8, FC3/FC4, F3/F4, and Fp1/Fp2, indicating increased right-hemispheric theta power. Significant increases from pre-stimulus presentation to resting-state were also observed at several sites, most notably at P7/P8 and F7/F8. The largest shift toward right-hemispheric dominance from resting-state to post-stimulus presentation occurred at FC3/FC4 and T7/T8.

In summary, hemispheric asymmetry, as indexed by the AI, was significantly modulated by timepoint across all frequency bands, with additional effects of electrode pair placement and age observed primarily in the alpha band. In the alpha band, asymmetry was predominantly left-hemispheric (negative AI) at posterior sites (e.g., P7/P8, T7/T8), especially during pre- and post-stimulus conditions, while frontal regions (e.g., F7/F8) showed increased right-hemispheric asymmetry (positive AI) post-stimulus. Age-related interactions revealed a dynamic modulation of alpha AI by cognitive state, with older adults showing greater right-hemispheric asymmetry at rest, which diminished during task engagement and partially re-emerged post-stimulus. In the beta band, AI increased significantly post-stimulus at frontal and frontocentral sites such as FC3/FC4 and F7/F8, indicating enhanced right-hemispheric engagement, while some parietal regions (e.g., P3/P4) shifted toward stronger left-hemispheric asymmetry following task completion. Both delta and theta bands showed robust increases in right-hemispheric dominance (positive AI) from rest to post-stimulus, especially at fronto-central and temporal sites (e.g., FC3/FC4, T7/T8), reflecting a task-induced asymmetry pattern. Overall, these results demonstrate that hemispheric asymmetry, as reflected in AI, is strongly influenced by frequency band, cognitive state, and electrode location, with distinct lateralization profiles emerging across different stages of task engagement.

To further explore lateralized brain activity, *t*-tests assessing hemispheric asymmetries were conducted. Medium effect sizes (Cohen’s *d*) indicating left-sided asymmetries were primarily observed in the alpha band during resting-state and post-stimulus in the PVT at parietal sites. Full results are presented in Supplementary material 7, and visual presentations are shown in Supplementary material 3 in Figure S31.

#### 3.1.2 Simon task

Results from the ANOVA revealed significant main effects of age, electrode pair, and timepoint, as well as several important interactions, suggesting that these factors significantly influenced EEG asymmetry across the frequency bands. Notably, the interactions between age and electrode pair, and electrode pair and timepoint were significant in several frequency bands. Only effects with at least medium effect sizes are described in the text; complete statistical details are provided in the corresponding Table 2; *post-hoc* analyses can be found in Supplementary material 1, separate ANOVAs for each electrode pair and *post-hoc* analyses in Supplementary material 2. A visual representation of effect sizes in the form of heatmaps can be found in Supplementary material 3 in Figure S2, sex-specific means in Supplementary material 4, and age-related visualizations of AI per frequency band in Supplementary material 3 in Figures S11–S14.

**Table 2:** ANOVA results for the Simon task in right-handed participants across frequency bands.

Parameter	*df*	*F*	*η*_p_^2^	*p*-value
Alpha (*N* = 430)				
Sex	1	0.066	<0.001	0.798
Age	1	13.955	0.038	<0.001***
Electrode pair	4.825	21.095	0.057	<0.001***
Sex * Electrode pair	4.825	1.639	0.005	0.149
Age * Electrode pair	4.825	9.195	0.026	<0.001***
Timepoint	1.606	79.032	0.183	<0.001***
Sex * Timepoint	1.606	2.049	0.006	0.140
Age * Timepoint	1.606	7.349	0.020	0.002**
Electrode pair * Timepoint	6.290	29.928	0.078	<0.001***
Sex * Electrode pair* Timepoint	6.290	2.121	0.006	0.045*
Age * Electrode pair * Timepoint	6.290	2.461	0.007	0.020*
Beta (*N* = 402)				
Sex	1	0.615	0.002	0.434
Age	1	2.814	0.009	0.094
Electrode pair	5.848	4.978	0.015	<0.001***
Sex * Electrode pair	5.848	1.303	0.004	0.253
Age * Electrode pair	5.848	3.078	0.009	<0.001***
Timepoint	1.181	37.494	0.104	<0.001***
Sex * Timepoint	1.181	1.543	0.005	0.217
Age * Timepoint	1.181	0.237	0.001	0.667
Electrode pair * Timepoint	5.680	9.284	0.028	<0.001***
Sex * Electrode pair * Timepoint	5.680	1.492	0.005	0.181
Age * Electrode pair * Timepoint	5.680	1.728	0.005	0.115
Delta (*N* = 378)				
Sex	1	2.879	0.010	0.091
Age	1	0.248	0.001	0.619
Electrode pair	6.569	5.747	0.019	<0.001***
Sex * Electrode pair	6.569	0.697	0.002	0.666
Age * Electrode pair	6.569	1.764	0.006	0.096
Timepoint	1.291	29.463	0.089	<0.001***
Sex * Timepoint	1.291	1.896	0.006	0.166
Age * Timepoint	1.291	1.622	0.005	0.205
Electrode pair * Timepoint	7.108	3.486	0.012	<0.001***
Sex * Electrode pair * Timepoint	7.108	0.391	0.001	0.910
Age * Electrode pair * Timepoint	7.108	1.010	0.003	0.423
Theta (*N* = 424)				
Sex	1	0.00014	<0.001	0.991
Age	1	4.891963	0.014	0.028*
Electrode pair	4.678	6.789645	0.019	<0.001***
Sex * Electrode pair	4.678	1.865	0.005	0.103
Age * Electrode pair	4.678	4.812	0.014	<0.001***
Timepoint	1.440	57.986	0.144	<0.001***
Sex * Timepoint	1.440	2.821	0.008	0.078*
Age * Timepoint	1.440	0.128	<0.001	0.811
Electrode pair * Timepoint	5.239	7.306	0.021	<0.001***
Sex * Electrode pair * Timepoint	5.239	1.793	0.005	0.108
Age * Electrode pair * Timepoint	5.239	1.218	0.004	0.297

*p < 0.05 (uncorrected), **p < 0.01, ***p < 0.001. The reported statistics include degrees of freedom (*df*), *F*-values (*F*), and *p*-values (*p*). Effect sizes are given as partial eta2 (*η*_p_^2^) and characterized according to Cohen (1988) as negligible: *η*_p_^2^ < 0.01, small: 0.01 ≤ *η*_p_^2^ < 0.06, medium: 0.06 ≤ *η*_p_^2^ < 0.14 (light green), or large: *η*_p_^2^ ≥ 0.14 (dark green). The sample size (*N*) is given in the table for each frequency band.

In the alpha frequency band, a significant main effect of timepoint was observed (*η*_p_^2^ = 0.183), along with a significant interaction between timepoint and electrode pair (*η*_p_^2^ = 0.078), indicating that asymmetry patterns varied across regions and task phases. *Post-hoc* analyses comparing resting, pre- and post-stimulus AIs separately for each electrode pair revealed that left-hemispheric asymmetry (more negative AI) was significantly greater pre- and post-stimulus compared to at rest at several posterior sites, including P7/P8, P3/P4, CP3/CP4, PO3/PO4, and O1/O2. The most pronounced shift occurred at P7/P8, where asymmetry decreased from strongly left-lateralized at rest to near zero post-stimulus, reflecting a substantial task-related reduction in asymmetry. In contrast, frontal regions such as F7/F8 exhibited increased right-hemispheric asymmetry during task engagement, highlighting region-specific lateralization responses to cognitive demands.

In the beta frequency band, timepoint had a significant main effect (*η*_p_^2^ = 0.104), indicating that hemispheric asymmetry varied across task phases. *Post-hoc* analyses comparing resting, pre- and post-stimulus AIs separately for each electrode pair revealed significantly greater right-hemispheric asymmetry (more positive AI) both pre- and post-stimulus compared to at rest at several electrode pairs, including P7/P8, F7/F8, FC3/FC4, and CP3/CP4. The most pronounced increase occurred at P7/P8, where AI shifted from strongly left-lateralized at rest to right-lateralized both pre- and post-stimulus. Conversely, some posterior sites such as P3/P4 and PO3/PO4 showed reduced asymmetry post-relative to pre-stimulus, suggesting a re-balancing or decline in asymmetry.

For the delta frequency band, timepoint had a significant main effect (*η*_p_^2^ = 0.089). *Post-hoc* analyses comparing resting, pre- and post-stimulus AIs separately for each electrode pair revealed significantly greater right-hemispheric asymmetry (more positive AI) both pre- and post-stimulus compared to at rest at several frontal and fronto-central electrode pairs, including F7/F8, FC3/FC4, and Fp1/Fp2. The strongest effect was observed at F7/F8, where AI values shifted from negative at rest to strongly positive post-stimulus, reflecting increased rightward asymmetry. A similar pattern was seen at P7/P8, with a reduction in leftward asymmetry during task conditions relative to rest.

In the theta frequency band, timepoint had a significant main effect (*η*_p_^2^ = 0.144). *Post-hoc* analyses comparing resting, pre- and post-stimulus AIs separately for each electrode pair revealed significantly greater right-hemispheric asymmetry (more positive AI) both pre- and post-stimulus compared to at rest at several electrode pairs, including F7/F8, P7/P8, and T7/T8. The largest increases in rightward asymmetry were observed at P7/P8 and F7/F8, with AI values shifting from negative at rest to positive pre- and post-stimulus. Additionally, a shift toward left-hemispheric asymmetry was observed at T7/T8, with significantly lower AI values post-compared to pre-stimulus.

In summary, across frequency bands (delta, theta, alpha, and beta), asymmetry patterns varied, showing both similarities and differences. In delta, right-sided asymmetry increased post-stimulus at FC3/FC4. In alpha, leftward asymmetry was stronger pre- and post-stimulus at P7/P8 and P3/P4. The theta band showed a rightward shift at F7/F8 and P7/P8, with some reduction in leftward asymmetry at T7/T8. In beta, rightward asymmetry increased at FC3/FC4 and F7/F8, though some regions, like P3/P4, returned to leftward asymmetry. Overall, right-sided asymmetry tended to increase post-stimulus, but each frequency band displayed distinct asymmetry dynamics.

To further explore lateralized brain activity, *t*-tests assessing hemispheric asymmetries were conducted. Full results are presented in Supplementary material 7, and visual presentations are shown in Supplementary material 3 in Figure S32.

#### 3.1.3 Stroop task

Only effects with at least medium effect sizes are described in the text; complete statistical details are provided in Table 3, *post-hoc* analyses can be found in Supplementary material 1, and separate ANOVAs for each electrode pair and *post-hoc* analyses in Supplementary material 2. A visual representation of effect sizes in the form of heatmaps can be found in Supplementary material 3 in Figure S3, sex-specific means in Supplementary material 4, and age-related visualizations of AIs per frequency band in Supplementary material 3 in Figures S15–S18.

**Table 3:** ANOVA results for the Stroop task in right-handed participants across frequency bands.

Parameter	*df*	*F*	*η*_p_^2^	*p*-value
Alpha (*N* = 407)				
Sex	1	0.007	<0.001	0.931
Age	1	10.760	0.032	0.001**
Electrode pair	4.629	20.342	0.058	<0.001***
Sex * Electrode pair	4.629	1.924	0.006	0.093
Age * Electrode pair	4.629	5.123	0.015	<0.001***
Timepoint	1.577	10.489	0.031	<0.001***
Sex * Timepoint	1.577	0.242	0.001	0.732
Age * Timepoint	1.577	6.836	0.020	0.003**
Electrode pair * Timepoint	5.846	15.667	0.046	<0.001***
Sex * Electrode pair * Timepoint	5.846	1.489	0.005	0.180
Age * Electrode pair * Timepoint	5.846	0.962	0.003	0.448
Beta (*N* = 390)				
Sex	1	2.223	0.007	0.137
Age	1	4.610	0.015	0.033*
Electrode pair	5.808	5.542	0.018	<0.001***
Sex * Electrode pair	5.808	1.056	0.003	0.387
Age * Electrode pair	5.808	0.886	0.003	0.502
Timepoint	1.206	3.707	0.012	0.047*
Sex * Timepoint	1.206	1.023	0.003	0.327
Age * Timepoint	1.206	0.655	0.002	0.446
Electrode pair * Timepoint	6.346	4.095	0.013	<0.001***
Sex * Electrode pair * Timepoint	6.346	0.723	0.002	0.639
Age * Electrode pair * Timepoint	6.346	0.990	0.003	0.433
Delta (*N* = 397)				
Sex	1	0.073	<0.001	0.788
Age	1	4.004	0.012	0.046*
Electrode pair	6.712	2.449	0.008	0.018*
Sex * Electrode pair	6.712	0.545	0.002	0.793
Age * Electrode pair	6.712	0.969	0.003	0.450
Timepoint	1.135	8.754	0.027	0.002**
Sex * Timepoint	1.135	0.052	<0.001	0.849
Age * Timepoint	1.135	0.512	0.002	0.497
Electrode pair * Timepoint	7.411	2.490	0.008	0.013*
Sex * Electrode pair * Timepoint	7.411	0.661	0.002	0.714
Age * Electrode pair * Timepoint	7.411	0.762	0.002	0.627
Theta (*N* = 417)				
Sex	1	0.183	0.001	0.669
Age	1	6.736	0.020	0.010**
Electrode pair	4.311	6.576	0.019	<0.001***
Sex * Electrode pair	4.311	1.472	0.004	0.204
Age * Electrode pair	4.311	2.596	0.008	0.031*
Timepoint	1.345	12.817	0.036	<0.001***
Sex * Timepoint	1.345	0.341	0.001	0.624
Age * Timepoint	1.345	0.383	0.001	0.599
Electrode pair * Timepoint	4.883	5.636	0.016	<0.001***
Sex * Electrode pair * Timepoint	4.883	0.916	0.003	0.468
Age * Electrode pair * Timepoint	4.883	0.806	0.002	0.543

*p < 0.05 (uncorrected), **p < 0.01, ***p < 0.001. The reported statistics include degrees of freedom (*df*), *F*-values (*F*), and *p*-values (*p*). Effect sizes are given as partial eta2 (*η*_p_^2^) and characterized according to Cohen (1988) as negligible: *η*_p_^2^ < 0.01, small: 0.01 ≤ *η*_p_^2^ < 0.06, medium: 0.06 ≤ *η*_p_^2^ < 0.14 (light green), or large: *η*_p_^2^ ≥ 0.14 (dark green). The sample size (*N*) is given in the table for each frequency band.

Across all frequency bands, no significant main effects or interactions reached a medium effect size (η_p_^2^ ≥ 0.06). While some effects were significant, their effect sizes remained small, indicating that the observed differences were not substantial in terms of practical significance (see Table 3).

To further explore lateralized brain activity, *t*-tests assessing hemispheric asymmetries were conducted. Full results are presented in Supplementary material 7, and visual presentations are shown in Supplementary material 3 in Figure S33.

### 3.2 Analyses including only non-right-handers

#### 3.2.1 Psychomotor vigilance task

The ANOVA results revealed significant main effects of age, electrode pair, and timepoint, along with significant interactions between age and electrode, and electrode pair and timepoint. These factors significantly influenced AI across frequency bands. Only effects with at least medium effect sizes are described in the text; complete statistical details are provided in the corresponding Table 4, *post-hoc* analyses can be found in Supplementary material 5, and separate ANOVAs for each electrode pair and *post-hoc* analyses in Supplementary material 6. A visual representation of effect sizes in the form of heatmaps can be found in Supplementary material 3 in Figure S4, sex-specific means in Supplementary material 4, and age-related visualizations of AIs per frequency band in Supplementary material 3 in Figures S19–S22.

**Table 4:** ANOVA results for the PVT in non-right-handed participants across frequency bands.

Parameter	*df*	*F*	*η*_p_^2^	*p*-value
Alpha (*N* = 109)				
Sex	1	1.717	0.020	0.194
Age	1	12.741	0.129	0.001**
Electrode pair	5.276	10.682	0.110	<0.001***
Sex * Electrode pair	5.276	2.08	0.024	0.063
Age * Electrode pair	5.276	2.71	0.031	0.018*
Timepoint	1.610	6.204	0.067	0.005**
Sex * Timepoint	1.610	3.657	0.041	0.037*
Age * Timepoint	1.610	1.399	0.016	0.249
Electrode pair * Timepoint	8.019	5.219	0.057	<0.001***
Sex * Electrode pair * Timepoint	8.019	1.876	0.021	0.061
Age * Electrode pair * Timepoint	8.019	0.686	0.008	0.704
Beta (*N* = 106)				
Sex	1	0.001	<0.001	0.978
Age	1	3.269	0.038	0.074
Electrode pair	6.007	0.648	0.008	0.692
Sex * Electrode pair	6.007	1.012	0.012	0.416
Age * Electrode pair	6.007	0.478	0.006	0.825
Timepoint	1.248	8.852	0.096	0.002**
Sex * Timepoint	1.248	1.535	0.018	0.222
Age * Timepoint	1.248	0.582	0.007	0.483
Electrode pair * Timepoint	6.419	2.683	0.031	0.012*
Sex * Electrode pair * Timepoint	6.419	1.360	0.016	0.225
Age * Electrode pair * Timepoint	6.419	1.060	0.013	0.387
Delta (*N* = 100)				
Sex	1	0.577	0.007	0.45
Age	1	0.999	0.013	0.321
Electrode pair	5.237	3.325	0.041	0.005**
Sex * Electrode pair	5.237	1.753	0.022	0.118
Age * Electrode pair	5.237	1.662	0.021	0.139
Timepoint	1.208	4.988	0.061	0.022*
Sex * Timepoint	1.208	0.059	0.001	0.854
Age * Timepoint	1.208	0.019	<0.001	0.926
Electrode pair * Timepoint	5.962	2.604	0.033	0.017*
Sex * Electrode pair * Timepoint	5.962	1.546	0.020	0.162
Age * Electrode pair * Timepoint	5.962	1.748	0.022	0.109
Theta (*N* = 104)				
Sex	1	0.101	0.001	0.752
Age	1	4.671	0.055	0.034*
Electrode pair	3.911	2.446	0.029	0.048*
Sex * Electrode pair	3.911	2.329	0.028	0.058
Age * Electrode pair	3.911	0.55	0.007	0.695
Timepoint	1.537	20.929	0.205	<0.001***
Sex * Timepoint	1.537	1.859	0.022	0.169
Age * Timepoint	1.537	0.342	0.004	0.654
Electrode pair * Timepoint	5.739	2.916	0.035	0.009**
Sex * Electrode pair * Timepoint	5.739	0.892	0.011	0.497
Age * Electrode pair * Timepoint	5.739	0.379	0.005	0.885

*p < 0.05 (uncorrected), **p < 0.01, ***p < 0.001. The reported statistics include degrees of freedom (*df*), *F*-values (*F*), and *p*-values (*p*). Effect sizes are given as partial eta2 (*η*_p_^2^) and characterized according to Cohen (1988) as negligible: *η*_p_^2^ < 0.01, small: 0.01 ≤ *η*_p_^2^ < 0.06, medium: 0.06 ≤ *η*_p_^2^ < 0.14 (light green), or large: *η*_p_^2^ ≥ 0.14 (dark green). The sample size (*N*) is given in the table for each frequency band.

For the alpha frequency band, electrode pair placement (*η*_p_^2^ = 0.110) and timepoint (*η*_p_^2^ = 0.067) had significant main effects. *Post-hoc* analyses comparing resting, pre- and post-stimulus AIs separately for each electrode pair revealed that AI for P7/P8 were significantly more negative both pre- and post-stimulus compared to at rest, indicating stronger left-hemispheric asymmetry. Similarly, T7/T8 showed greater left-hemispheric asymmetry post-stimulus relative to at rest. A significant main effect was also evident for age (*η*_p_^2^ = 0.129), indicating that alpha AI shifted positively with increasing age across all conditions (resting-state, pre- and post-stimulus), suggesting a general trend toward greater right-hemispheric alpha power in older non-right-handed individuals. This age-related increase in rightward asymmetry was most prominent post-stimulus, particularly at frontal and parietal electrode pairs (e.g., F7/F8, P3/P4), where slopes were consistently positive (see Supplementary material 3 in Figure S19).

In the beta frequency band, timepoint had a significant main effect (*η*_p_^2^ = 0.096). *Post-hoc* analyses comparing resting, pre- and post-stimulus AIs separately for each electrode pair revealed that AI were significantly higher post-stimulus compared to resting-state for electrode pairs FC3/FC4, P7/P8, and F3/F4, indicating a shift toward increased asymmetry after task completion. Specifically, FC3/FC4 showed a marked increase in right-hemispheric asymmetry post-stimulus, while P7/P8 and F3/F4 exhibited stronger left-hemispheric asymmetry in the post-stimulus condition.

For the delta frequency band, timepoint showed a significant main effect (*η*_p_^2^ = 0.061). *Post-hoc* analyses indicated that AI were significantly higher post-stimulus compared to at rest for the electrode pair CP3/CP4, reflecting increased right-hemispheric asymmetry following task engagement.

In the theta frequency band, timepoint had a significant main effect (*η*_p_^2^ = 0.205). *Post-hoc* analyses comparing resting, pre- and post-stimulus AIs separately for each electrode pair revealed significant changes in AI across multiple electrode pairs. Specifically, T7/T8 exhibited a marked increase in right-hemispheric asymmetry post-stimulus compared to both pre-stimulus and at rest. Similarly, F7/F8 and P7/P8 also showed significant increases in AI post-stimulus relative to at rest, indicating localized lateralization effects at these sites. The most pronounced shifts were observed in T7/T8, highlighting the importance of this region in task-related asymmetry.

In summary, across frequency bands, significant changes in AI were observed at various electrode pairs, with timepoint consistently showing a main effect. In the alpha band, shifts in asymmetry were most prominent at posterior regions, while frontal and frontocentral areas exhibited more rightward asymmetry. The beta band revealed localized changes in asymmetry at FC3/FC4, P7/P8, and F3/F4, particularly in the post-stimulus period. In the delta band, small but significant shifts in asymmetry were noted at CP3/CP4 post-stimulus. The theta band showed prominent right-hemispheric asymmetry at T7/T8, F7/F8, and P7/P8 both post- and pre-stimulus. Overall, these findings highlight frequency-dependent patterns of asymmetry changes in response to cognitive tasks, with posterior and temporal regions showing the most pronounced effects.

To further explore lateralized brain activity, *t*-tests assessing hemispheric asymmetries were conducted. Medium effect sizes (Cohen’s *d*) indicating left-sided asymmetries were primarily observed in the alpha band during resting-state and post-stimulus in the PVT at parietal sites. These asymmetries were found in the alpha, delta, and theta frequency bands, with electrode pairs P3/P4, P7/P8, and CP3/CP4, PO3/PO4. Full results are presented in Supplementary material 7, and visual presentations are shown in Supplementary material 3 in Figure 34.

#### 3.2.2 Simon task

The ANOVA results revealed significant main effects of age, electrode pair, and timepoint, with interactions between age and electrode pair and electrode pair and timepoint. These factors significantly influenced EEG activity across the frequency bands. Only effects with at least medium effect sizes are described in the text; complete statistical details are provided in the corresponding Table 5, *post-hoc* analyses can be found in Supplementary material 5, and separate ANOVAs for each electrode pair and *post-hoc* analyses in Supplementary material 6. A visual representation of effect sizes in the form of heatmaps can be found in Supplementary material 3 in Figure S5, sex-specific means in Supplementary material 4, and age-related visualizations of AIs per frequency band in Supplementary material 3 in Figures S23–S26.

**Table 5:** ANOVA results for the Simon task in non-right-handed participants across frequency bands.

Parameter	*df*	*F*	*η*_p_^2^	*p*-value
Alpha (*N* = 112)				
Sex	1	1.871	0.021	0.175
Age	1	11.254	0.112	0.001**
Electrode pair	4.956	4.643	0.050	<0.001***
Sex * Electrode pair	4.956	1.313	0.015	0.258
Age * Electrode pair	4.956	3.438	0.037	0.005**
Timepoint	1.573	24.738	0.217	<0.001***
Sex * Timepoint	1.573	1.41	0.016	0.247
Age * Timepoint	1.573	0.97	0.011	0.364
Electrode pair * Timepoint	6.703	10.878	0.109	<0.001***
Sex * Electrode pair * Timepoint	6.703	1.676	0.018	0.115
Age * Electrode pair * Timepoint	6.703	1.438	0.016	0.190
Beta (*N* = 104)				
Sex	1	0.42	0.005	0.519
Age	1	10.286	0.113	0.002**
Electrode pair	5.796	2.469	0.030	0.025*
Sex * Electrode pair	5.796	0.375	0.005	0.890
Age * Electrode pair	5.796	1.477	0.018	0.187
Timepoint	1.234	20.952	0.206	<0.001***
Sex * Timepoint	1.234	0.593	0.007	0.477
Age * Timepoint	1.234	1.934	0.023	0.165
Electrode pair * Timepoint	5.657	2.722	0.033	0.015*
Sex * Electrode pair * Timepoint	5.657	1.717	0.021	0.120
Age * Electrode * Timepoint	5.657	1.469	0.018	0.191
Delta (*N* = 96)				
Sex	1	0.436	0.006	0.511
Age	1	5.065	0.065	0.027*
Electrode pair	5.993	1.237	0.017	0.286
Sex * Electrode pair	5.993	0.582	0.008	0.745
Age * Electrode pair	5.993	0.933	0.013	0.471
Timepoint	1.319	8.491	0.104	0.002**
Sex * Timepoint	1.319	0.236	0.003	0.695
Age * Timepoint	1.319	1.199	0.016	0.290
Electrode pair * Timepoint	6.108	0.756	0.010	0.607
Sex * Electrode pair * Timepoint	6.108	0.397	0.005	0.883
Age * Electrode pair * Timepoint	6.108	1.052	0.014	0.392
Theta (*N* = 111)				
Sex	1	0.938	0.011	0.335
Age	1	6.671	0.070	0.011*
Electrode pair	4.157	2.239	0.025	0.062
Sex * Electrode pair	4.157	1.903	0.021	0.107
Age * Electrode pair	4.157	1.141	0.013	0.337
Timepoint	1.309	25.875	0.227	<0.001***
Sex * Timepoint	1.309	0.124	0.001	0.793
Age * Timepoint	1.309	0.531	0.006	0.514
Electrode pair * Timepoint	4.678	3.567	0.039	0.004**
Sex * Electrode pair * Timepoint	4.678	0.35	0.004	0.871
Age * Electrode pair * Timepoint	4.678	1.03	0.012	0.397

*p < 0.05 (uncorrected), **p < 0.01, ***p < 0.001. The reported statistics include degrees of freedom (*df*), *F*-values (*F*), and *p*-values (*p*). Effect sizes are given as partial eta2 (*η*_p_^2^) and characterized according to Cohen (1988) as negligible: *η*_p_^2^ < 0.01, small: 0.01 ≤ *η*_p_^2^ < 0.06, medium: 0.06 ≤ *η*_p_^2^ < 0.14 (light green), or large: *η*_p_^2^ ≥ 0.14 (dark green). The sample size (*N*) is given in the table for each frequency band.

In the alpha frequency band, age demonstrated a significant effect (*η*_p_^2^ = 0.112) with alpha AI increasing across all conditions, resting-state, pre-stimulus, and post-stimulus, indicating a general age-related shift toward greater right-hemispheric alpha power in non-right-handed individuals. This age-related increase in rightward asymmetry was most prominent post-stimulus, particularly at frontal and parietal electrode pairs (e.g., F7/F8, P3/P4), where regression slopes were consistently positive, indicating a directional shift toward enhanced right-hemispheric alpha asymmetry with age (see Supplementary material 3 in Figure S23).

Timepoint showed a significant main effect (*η*_p_^2^ = 0.217), with significant interactions between electrode pair and timepoint (*η*_p_^2^ = 0.109). *Post-hoc* analyses comparing resting, pre- and post-stimulus AIs separately for each electrode pair revealed significant shifts in AI across electrode pairs, particularly at P7/P8 and T7/T8. At P7/P8, leftward asymmetry at rest decreased significantly towards zero post-stimulus, indicating a reduction in left-hemispheric dominance. Conversely, at T7/T8, rightward asymmetry increased both pre- and post-stimulus compared to at rest.

In the beta frequency band, age had a significant effect (*η*_p_^2^ = 0.113), indicating that beta AI shifted positively with increasing age across all conditions (resting-state, pre- and post-stimulus), suggesting a general trend toward greater right-hemispheric power in older non-right-handed individuals. This age-related increase in rightward asymmetry was most prominent post-stimulus, particularly in temporal electrode pairs (T7/T8), where slopes were consistently positive (see Supplementary material 3 in Figure S24). Moreover, timepoint yielded a significant main effect (*η*_p_^2^ = 0.206); *post-hoc* analyses comparing resting, pre- and post-stimulus AIs separately for each electrode pair showed significant increases in right-hemispheric asymmetry (more positive AI values) at P7/P8 and T7/T8 both pre- and post-stimulus compared to at rest. Specifically, AI at P7/P8 shifted from left- at resting-state to strongly right-sided post-stimulus, while T7/T8 showed a comparable shift toward right-hemispheric dominance.

For the delta frequency band, age showed a significant main effect (*η*_p_^2^ = 0.065), with a similar trend as seen in the beta frequency band (see Supplementary material 3 in Figure S25). Additionally, a significant effect of timepoint was observed (*η*_p_^2^ = 0.104); *post-hoc* analyses comparing resting, pre- and post-stimulus AIs separately for each electrode pair showed significantly increased right-hemispheric asymmetry at F7/F8 both pre- and post-stimulus compared to at rest. AI shifted from negative at resting-state to positive during the task, indicating enhanced rightward asymmetry in delta power with cognitive engagement.

In the theta frequency band, age showed a significant effect (*η*_p_^2^ = 0.070), with a trend towards increased right-hemispheric dominance with increasing age that was most pronounced pre-stimulus at temporal electrode pairs (see Supplementary material 3 in Figure S26). Timepoint had a significant main effect (*η*_p_^2^ = 0.227); *post-hoc* analyses comparing resting, pre- and post-stimulus AIs separately for each electrode pair revealed increased right-hemispheric asymmetry both pre- and post-stimulus compared to at rest at T7/T8, P7/P8, and F7/F8. The strongest shift occurred at T7/T8 from resting-state to pre-stimulus.

In summary, results show that age is associated with increased right-hemispheric asymmetry across all frequency bands in non-right-handed individuals. Significant shifts in asymmetry were observed in the alpha, beta, delta, and theta bands, with the most pronounced changes occurring post-stimulus. Temporal regions (T7/T8) consistently showed increased right-sided asymmetry, while leftward asymmetry (e.g., P7/P8) decreased. Timepoint effects highlighted dynamic changes in hemispheric asymmetry, with enhanced right-hemispheric activity during task conditions compared to resting-state.

To further explore lateralized brain activity, *t*-tests assessing hemispheric asymmetries were conducted. Full results are presented in Supplementary material 7, and visual presentations are shown in Supplementary material 3 in Figure S35.

#### 3.2.3 Stroop task

The ANOVA results revealed significant main effects of age, electrode pair, and timepoint, with interactions between age and electrode pair and electrode pair and timepoint. These factors significantly influenced AI across frequency bands. Only effects with at least medium effect sizes are described in the text; complete statistical details are provided in the corresponding Table 6, *post-hoc* analyses can be found in Supplementary material 5, and separate ANOVAs for each electrode pair and *post-hoc* analyses in Supplementary material 6. A visual representation of effect sizes in the form of heatmaps can be found in Supplementary material 3 in Figure S6, sex-specific means in Supplementary material 4, and age-related visualizations of AIs per frequency band in Supplementary material 3 in Figures S27–S30.

**Table 6:** ANOVA results for the Stroop task in non-right-handed participants across frequency bands.

Parameter	*df*	*F*	*η*_p_^2^	*p*-value
Alpha (*N* = 105)				
Sex	1	1.229	0.015	0.271
Age	1	4.26	0.049	0.042*
Electrode pair	4.25	5.199	0.060	<0.001***
Sex * Electrode pair	4.25	1.153	0.014	0.332
Age * Electrode pair	4.25	2.505	0.030	0.039*
Timepoint	1.331	3.816	0.044	0.042*
Sex * Timepoint	1.331	0.288	0.003	0.659
Age * Timepoint	1.331	0.446	0.005	0.561
Electrode pair * Timepoint	5.706	3.011	0.035	0.008**
Sex * Electrode pair * Timepoint	5.706	0.523	0.006	0.782
Age * Electrode pair * Timepoint	5.706	0.544	0.007	0.766
Beta (*N* = 93)				
Sex	1	1.069	0.015	0.305
Age	1	0.022	<0.001	0.883
Electrode pair	5.328	2.389	0.033	0.034*
Sex * Electrode pair	5.328	0.994	0.014	0.424
Age * Electrode pair	5.328	1.712	0.024	0.126
Timepoint	1.303	0.698	0.010	0.442
Sex * Timepoint	1.303	2.196	0.030	0.135
Age * Timepoint	1.303	4.614	0.062	0.025*
Electrode pair * Timepoint	5.728	1.54	0.022	0.167
Sex * Electrode pair * Timepoint	5.728	0.612	0.009	0.713
Age * Electrode pair * Timepoint	5.728	1.408	0.020	0.213
Delta (*N* = 100)				
Sex	1	0.349	0.004	0.556
Age	1	0.296	0.004	0.588
Electrode pair	6.751	2.42	0.030	0.021*
Sex * Electrode pair	6.751	1.184	0.015	0.311
Age * Electrode pair	6.751	0.371	0.005	0.915
Timepoint	1.118	13.21	0.145	<0.001***
Sex * Timepoint	1.118	0.017	<0.001	0.917
Age * Timepoint	1.118	0.523	0.007	0.491
Electrode pair * Timepoint	6.938	1.29	0.016	0.254
Sex * Electrode pair * Timepoint	6.938	0.664	0.008	0.701
Age * Electrode pair * Timepoint	6.938	0.724	0.009	0.650
Theta (*N* = 106)				
Sex	1	0.27	0.003	0.605
Age	1	1.563	0.018	0.215
Electrode pair	3.514	1.989	0.023	0.105
Sex * Electrode pair	3.514	1.846	0.022	0.129
Age * Electrode pair	3.514	0.911	0.011	0.448
Timepoint	1.253	3.431	0.040	0.057
Sex * Timepoint	1.253	0.205	0.002	0.708
Age * Timepoint	1.253	0.743	0.009	0.419
Electrode pair * Timepoint	4.355	1.702	0.020	0.143
Sex * Electrode pair * Timepoint	4.355	0.521	0.006	0.736
Age * Electrode pair * Timepoint	4.355	0.244	0.003	0.925

*p < 0.05 (uncorrected), **p < 0.01, ***p < 0.001. The reported statistics include degrees of freedom (df), *F*-values (*F*), and *p*-values (*p*). Effect sizes are given as partial eta2 (*η*_p_^2^) and characterized according to Cohen (1988) as negligible: *η*_p_^2^ < 0.01, small: 0.01 ≤ *η*_p_^2^ < 0.06, medium: 0.06 ≤ *η*_p_^2^ < 0.14 (light green), or large: *η*_p_^2^ ≥ 0.14 (dark green). The sample size (*N*) is given in the table for each frequency band.

In the alpha frequency band, electrode pair placement significantly influenced alpha asymmetry (*η*_p_^2^ = 0.060). *Post-hoc* analyses comparing resting, pre- and post-stimulus AIs separately for each electrode pair revealed a pronounced reduction in left-hemispheric asymmetry at posterior sites, particularly P7/P8 and P3/P4, at rest compared to both pre- and post-stimulus. Conversely, F7/F8 exhibited a shift toward greater right-hemispheric asymmetry during the task.

In the beta frequency band, a significant age × timepoint interaction (*η*_p_^2^ = 0.062) was observed, with the Fp1/Fp2 electrode pair showing more negative AI with increasing age, particularly pre- and post-stimulus. While the resting-state showed near-zero asymmetry (indicating balanced hemispheric activity), older individuals displayed enhanced left-hemispheric dominance (negative AI) during task conditions. This suggests that age-related changes in hemispheric asymmetry are most prominent at Fp1/Fp2 during cognitive tasks, with no similar patterns observed at other electrode pairs (see Supplementary material 3 in Figure S28).

In the delta frequency band, timepoint demonstrated a significant main effect (*η*_p_^2^ = 0.145); however, no effects survived correction for multiple comparisons in the *post-hoc* tests. In the theta frequency band, no effect reached a medium effect size.

In summary, across frequency bands, asymmetry patterns exhibited both shared trends and distinct dynamics. In the delta band, rightward asymmetry increased post-stimulus at FC3/FC4. In alpha, leftward asymmetry was more pronounced at P7/P8 and P3/P4 pre- and post-stimulus. The theta band showed a rightward shift at F7/F8 and P7/P8, with some reduction in leftward asymmetry at T7/T8. In beta, rightward asymmetry increased at FC3/FC4 and F7/F8, while regions like P3/P4 returned to leftward asymmetry. Overall, post-stimulus periods generally favored rightward asymmetry.

To further explore lateralized brain activity, *t*-tests assessing hemispheric asymmetries were conducted. Full results are presented in Supplementary material 7, and visual presentations are shown in Supplementary material 3 in Figure S36.

### 3.3 Correlation between frequency bands

Pearson’s correlations were calculated separately for each task, comparing AI across frequency bands for each electrode pair for pre- and post-stimulus periods, as well as for resting-state. The results revealed significant positive correlations between frequency bands across all electrode pairs. Pearson’s *r* effect sizes are categorized as small (0.1 ≤ *r* < 0.3), medium (0.3 ≤ *r* < 0.5), or large (*r* ≥ 0.5).

**Figure 2:**
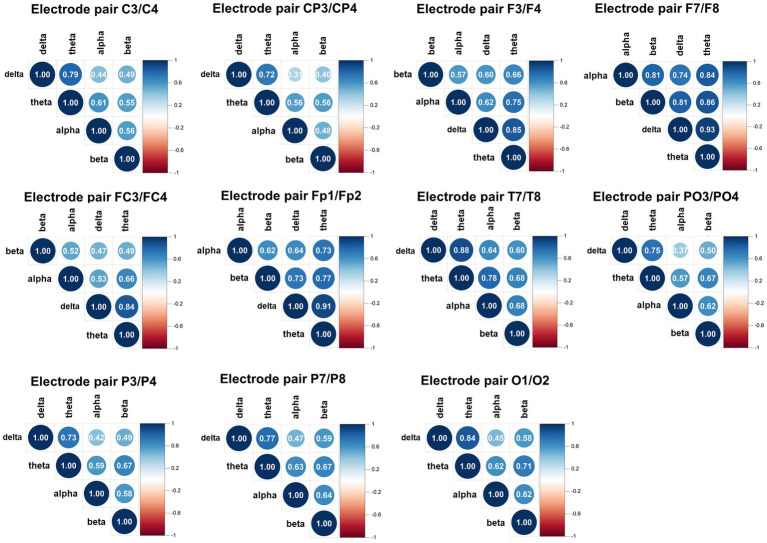
Correlation matrices of Asymmetry Indexes between frequency bands for each electrode pair for the resting-state data. The correlations between different frequency bands were high across all electrodes (*r* = 0.31–0.91), with highest correlations over frontal regions (Fp1/Fp2: *r* = 0.62–0.91 and F7/F8: *r* = 0.74–0.93), and weakest correlations over central (C3/C4: *r* = 0.44–0.79), centroparietal (CP3/CP4: *r* = 0.31–0.72), parietal (P3/P4: *r* = 0.42–0.73), and parietooccipital (PO3/PO4: *r* = 0.45–0.75) areas. Regarding the different dyads of frequency bands, theta power exhibited high correlations with all other frequency bands (delta: *r* = 0.72–0.93, alpha: *r* = 0.56–0.84, beta: *r* = 0.49–0.86), especially with delta power. Delta power itself, however, was only weakly correlated with the faster frequency bands (alpha: *r* = 0.31–0.74 and beta: *r* = 0.40–0.81), showing the smallest correlations with alpha power. Correlations between alpha and beta were overall of medium effect size (*r* = 0.48–0.81).

The correlation analysis of resting-state power across electrode pairs presented varying degrees of interhemispheric connectivity across frequency bands (Figure 2). Overall, the correlations between different frequency bands were high across all electrodes (*r* = 0.31–0.91), with highest correlations over frontal regions (Fp1/Fp2: *r* = 0.62–0.91 and F7/F8: *r* = 0.74–0.93), and weakest correlations over central (C3/C4: *r* = 0.44–0.79), centroparietal (CP3/CP4: *r* = 0.31–0.72), parietal (P3/P4: *r* = 0.42–0.73), and parietooccipital (PO3/PO4: *r* = 0.45–0.75) areas. Regarding the different dyads of frequency bands, theta power exhibited high correlations with all other frequency bands (delta: *r* = 0.72–0.93, alpha: *r* = 0.56–0.84, beta: *r* = 0.49–0.86), especially with delta power. Delta power itself, however, was only weakly correlated with the faster frequency bands (alpha: *r* = 0.31–0.74 and beta: *r* = 0.40–0.81), showing the smallest correlations with alpha power. Correlations between alpha and beta were overall of medium effect size (*r* = 0.48–0.81).

**Figure 3:**
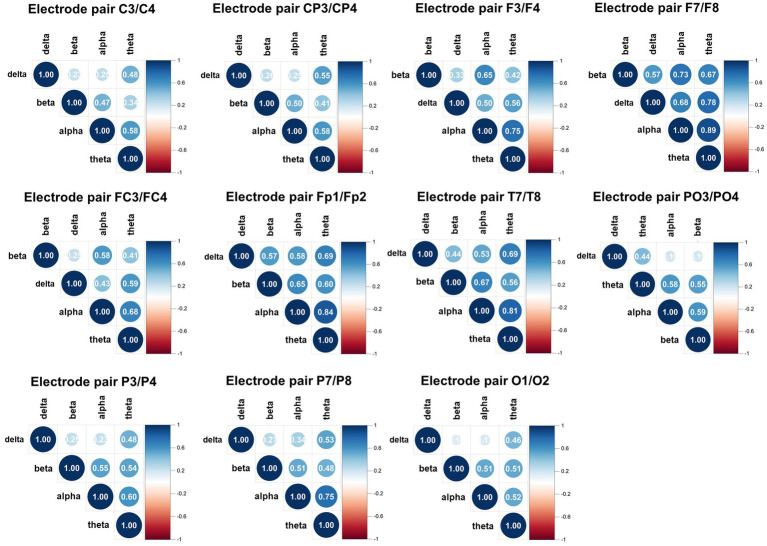
Correlation matrices of Asymmetry Indexes between frequency bands for each electrode pair for pre-stimulus data in the Psychomotor Vigilance Task (PVT). Strongest correlations were observed between the alpha and theta bands, especially over frontal (F7/F8 and Fp1/Fp2) and temporal (T7/T8) regions. Correlations between alpha and beta (*r* = 0.47–0.73) and between theta and delta (*r* = 0.44–0.78) were of medium to large effect size across all electrodes. Notably, the correlation between theta and delta was reduced at all electrode sites pre-stimulus presentation compared to at rest. This reduction in correlation during the PVT task compared to at resting-state was also found for the correlation between delta and alpha, as well as between delta and beta frequency bands.

The correlation analysis of AI pre-stimulus in the PVT showed distinct interhemispheric connectivity patterns across frequency bands, with strong correlations noted in frontal regions, particularly over F7/F8 (*r* = 0.57–0.89) and Fp1/Fp2 (*r* = 0.57–0.84). The strongest correlations were observed between the alpha and theta bands, especially over frontal (F7/F8 and Fp1/Fp2) and temporal (T7/T8) regions. Correlations between alpha and beta (*r* = 0.47–0.73) and between theta and delta (*r* = 0.44–0.78) were of medium to large effect size across all electrodes. Notably, the correlation between theta and delta was reduced at all electrode sites pre-stimulus presentation compared to at rest. This reduction in correlation during the PVT task compared to at resting-state was also found for the correlation between delta and alpha, as well as between delta and beta frequency bands (Figure 3).

**Figure 4:**
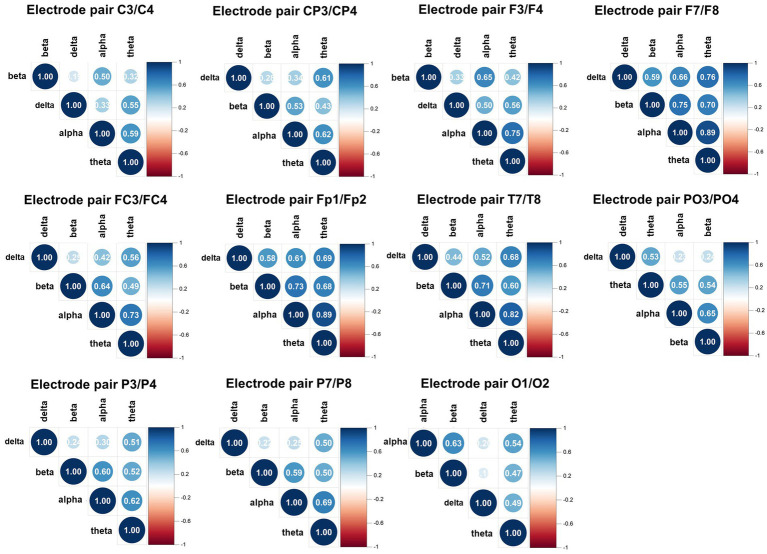
Correlation matrices of Asymmetry Indexes between frequency bands for each electrode pair for the post-stimulus periods in the Psychomotor Vigilance Task (PVT). Strong correlations were evident over frontal regions, particularly over F7/F8 (*r* = 0.56–0.89) and Fp1/Fp2 (*r* = 0.58–0.89). The overall pattern of correlations between different frequency bands was the same as during pre-stimulus, showing largest correlations between alpha and theta (*r* = 0.54–0.89), followed by correlations between alpha and beta (*r* = 0.53–0.75), and between beta and theta (*r* = 0.49–0.76) across all electrodes.

As before, the correlation analysis of AI across all frequency bands for the post-stimulus period in the PVT displayed strong correlations over frontal regions, particularly over F7/F8 (*r* = 0.56–0.89) and Fp1/Fp2 (*r* = 0.58–0.89). The overall pattern of correlations between different frequency bands was the same as during pre-stimulus, showing the largest correlations between alpha and theta (*r* = 0.54–0.89), followed by correlations between alpha and beta (*r* = 0.53–0.75), and between beta and theta (*r* = 0.49–0.76) across all electrodes (Figure 4).

**Figure 5:**
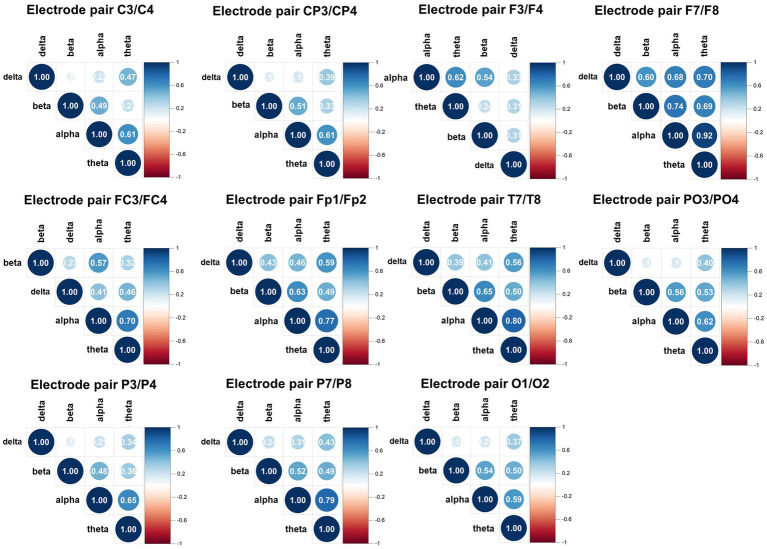
Correlation matrices of Asymmetry Indexes between frequency bands for each electrode pair for the pre-stimulus periods in the Simon Task. Highest correlations were found over frontal regions, especially over F7/F8 (*r* = 0.60–0.92). Strongest correlations were found between alpha and theta bands (*r* = 0.59–0.92), especially over F7/F8 (*r* = 0.92) and T7/T8 (*r* = 0.80). Correlations between alpha and beta bands were mostly of large size (*r* = 0.48–0.74), and correlations between theta and delta were mostly of medium size (*r* = 0.31–0.70) across all electrodes. Smallest correlations were found between beta and delta (*r* = 0.10–0.60), followed by correlations between alpha and delta (*r* = 0.19–0.68), as well as between beta and theta (*r* = 0.23–0.69) frequency bands.

The correlation analysis of AI across frequency bands for the pre-stimulus periods in the Simon task revealed a similar pattern to the pre-stimulus data of the PVT task (see Figure 5). The highest correlations were found over frontal regions, especially over F7/F8 (*r* = 0.60–0.92). The strongest correlations were found between alpha and theta bands (*r* = 0.59–0.92), especially over F7/F8 (*r* = 0.92) and T7/T8 (*r* = 0.80). Correlations between alpha and beta bands were mostly of large size (*r* = 0.48–0.74), and correlations between theta and delta were mostly of medium size (*r* = 0.31–0.70) across all electrodes. The weakest correlations were found between beta and delta (*r* = 0.10–0.60), followed by correlations between alpha and delta (*r* = 0.19–0.68), as well as between beta and theta (*r* = 0.23–0.69) frequency bands.

**Figure 6:**
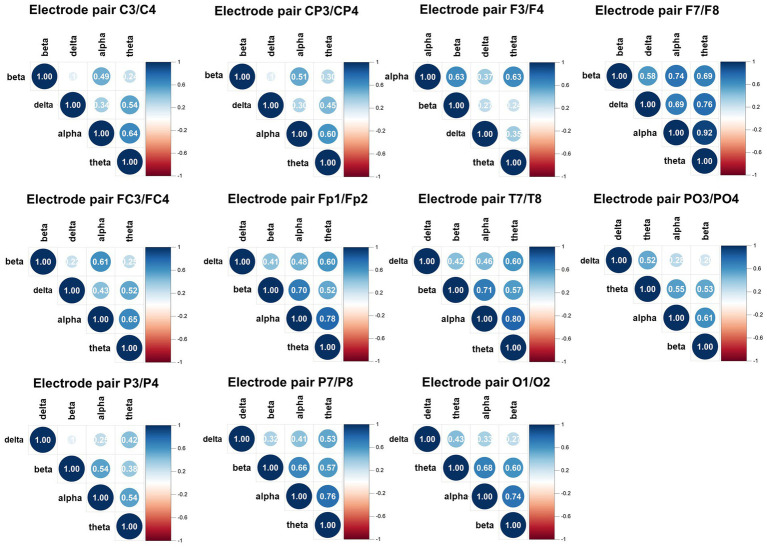
Correlation matrices of Asymmetry Indexes between frequency bands for each electrode pair for the post-stimulus periods in the Simon Task. Highest correlations were found over frontal (F7/F8: *r* = 0.58–0.92) regions, with strongest coupling between alpha and beta bands (*r* = 0.92). As before, strongest correlations across all electrodes were found for alpha and theta (*r* = 0.54–0.92), followed by alpha and beta (*r* = 0.49–0.74), as well as theta and delta (*r* = 0.35–0.76). Weakest correlations were found between beta and delta (*r* = 0.11–0.58), followed by correlations between alpha and delta (*r* = 0.25–0.69), and between beta and theta (*r* = 0.24–0.69). This pattern suggests higher interhemispheric coupling for frequency bands with more similar frequencies.

The correlation analysis of AI across frequency bands for the post-stimulus periods in the Simon task showed comparable results to the pre-stimulus data. Highest correlations were found over frontal (F7/F8: *r* = 0.58–0.92) regions, with the strongest coupling between alpha and beta bands (*r* = 0.92). As before, the strongest correlations across all electrodes were found for alpha and theta (*r* = 0.54–0.92), followed by alpha and beta (*r* = 0.49–0.74), as well as theta and delta (*r* = 0.35–0.76). The weakest correlations were found between beta and delta (*r* = 0.11–0.58), followed by correlations between alpha and delta (*r* = 0.25–0.69), and between beta and theta (*r* = 0.24–0.69). This pattern suggests higher interhemispheric coupling for frequency bands with more similar frequencies (Figure 6).

**Figure 7:**
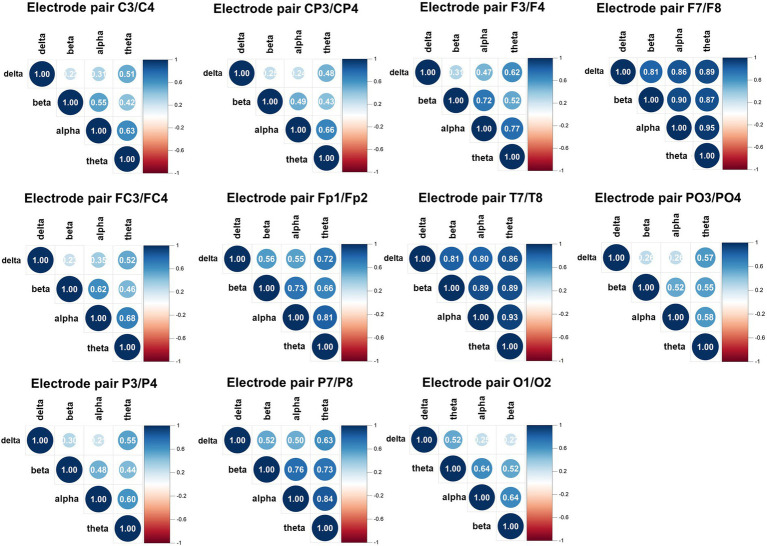
Correlation matrices of Asymmetry Indexes between frequency bands for each electrode pair for the pre-stimulus periods in the Stroop Task. The strongest correlations were evident in frontal and temporal regions (F7/F8, T7/T8: r ≥ 0.8) across all frequency bands, with the strongest correlation between alpha and theta frequency bands (r > 0.92). The weakest correlations were observed at specific sites within frontal (F3/F4, FC3/FC4), central (C3/C4), centroparietal (CP3/CP4), and occipital/parieto-occipital (PO3/PO4, O1/O2) regions for interactions between delta and beta as well as delta and alpha frequency bands.

The correlation analysis of AI across frequency bands for pre-stimulus data in the Stroop task revealed the strongest correlations in frontal and temporal regions (F7/F8, T7/T8: r ≥ 0.8) across all frequency bands, with the strongest correlation between alpha and theta frequency bands (r > 0.92). The weakest correlations were observed at specific sites within frontal (F3/F4, FC3/FC4), central (C3/C4), centroparietal (CP3/CP4), and occipital/parieto-occipital (PO3/PO4, O1/O2) regions for interactions between delta and beta as well as delta and alpha frequency bands (see Figure 7). Notably, these weaker delta-alpha and delta-beta correlations were specific to a subset of electrodes, indicating regionally restricted reductions in cross-frequency coupling involving the delta band.

**Figure 8:**
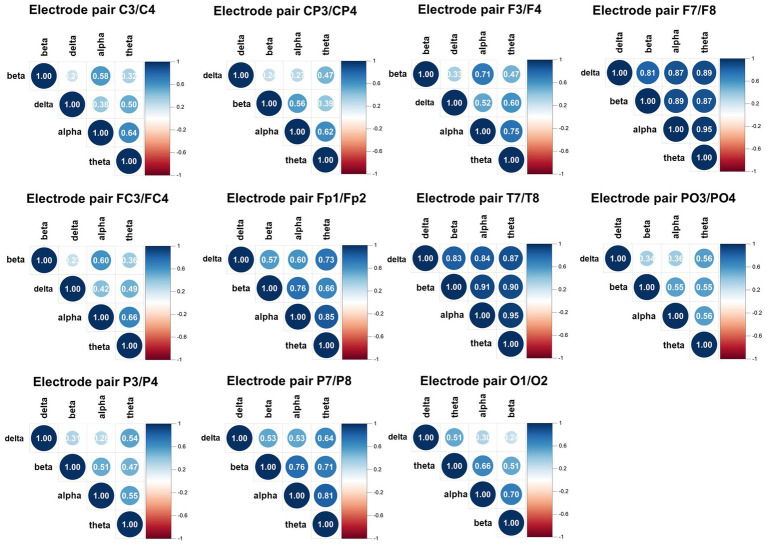
Correlation matrices of Asymmetry Indexes between frequency bands for each electrode pair for post-stimulus periods in the Stroop Task. Strongest correlations were evident over frontal and temporal regions (F7/F8, T7/T8; r > 0.8) across all frequency bands, with the highest correlation observed between alpha and theta bands (*r* = 0.95). In contrast, the weakest correlations were found at central (C3/C4), frontocentral (FC3/FC4), centroparietal (CP3/CP4), parietal (P3/P4), parieto-occipital (PO3/PO4), and occipital (O1/O2) sites, particularly for interactions between delta and other frequency bands.

The correlation analysis of AI across frequency bands for the post-stimulus periods in the Stroop task revealed the strongest correlations over frontal and temporal regions (F7/F8, T7/T8; *r* > 0.8) across all frequency bands, with the highest correlation observed between alpha and theta bands (*r* = 0.95). In contrast, the weakest correlations were found at central (C3/C4), frontocentral (FC3/FC4), centroparietal (CP3/CP4), parietal (P3/P4), parieto-occipital (PO3/PO4), and occipital (O1/O2) sites, particularly for interactions between delta and other frequency bands (see Figure 8).

The degree of correlation varied systematically by frequency band and region across all conditions. Frontal areas consistently demonstrated strong coupling during both rest and task performance. This coupling was enhanced post-stimulus compared to pre-stimulus. In contrast, central, parietal, and occipital regions exhibited weaker connectivity, especially for interactions involving the delta band. Additionally, task-specific patterns emerged: the PVT displayed stable correlations across phases, consistent with sustained attention. The Simon task revealed increased post-stimulus alpha-beta correlations, while the Stroop task showed the strongest alpha-theta correlations.

## 4 Discussion

Hemispheric asymmetries are often altered in clinical groups (Ocklenburg et al., 2024, 2025; Packheiser et al., 2025), and EEG is an important technique to assess altered hemispheric asymmetries in different diagnoses. In particular, EEG alpha band asymmetries have been investigated in many clinical groups (Meyer et al., 2018; Nusslock et al., 2018; Smith et al., 2018). However, even in non-clinical groups, the mechanisms underlying EEG asymmetries are still poorly understood.

The present study aimed to explore how alpha, beta, delta, and theta band asymmetries vary across tasks with differing motor and cognitive demands in a large sample of healthy adults. EEG activity was analyzed during three tasks with varying degrees of hand involvement: a PVT (dominant hand), a Simon task (both hands), and a Stroop task (both hands). Resting-state asymmetries were compared to task-related asymmetries to descriptively assess dynamic changes from baseline, without assuming directional or frequency-specific effects. Individual factors such as age, sex, and handedness were considered descriptively to capture interindividual variability in functional lateralization. The results highlight that EEG asymmetry patterns vary across tasks and individuals, with some tasks (e.g., Stroop) showing no significant changes, emphasizing the context-dependent and exploratory nature of these observations.

Inter-frequency correlations of AI varied systematically across tasks, frequency bands, electrode pairs, and temporal conditions (pre-stimulus, post-stimulus, and resting state). Overall, frontal regions showed comparatively stronger correlations across conditions, whereas central, parietal, and occipital regions exhibited weaker and more variable correlations, particularly for lower-frequency interactions. Task-specific patterns further suggested differential modulation of coupling strength, with more stable correlations during the PVT, increased post-stimulus alpha-beta coupling during the Simon task, and pronounced alpha-theta correlations during the Stroop task. These differences might reflect task-dependent modulation of functional interactions between frequency bands, potentially linked to varying demands on sustained attention, response selection, and executive control.

Task-related asymmetries differed in some but not all measures from resting-state patterns, indicating a dynamic component that is task- and time-dependent. Additionally, motor execution appeared to contribute more than motor preparation under certain conditions (e.g., PVT), though cognitive contributions cannot be ruled out. The study also found that age and handedness influenced asymmetry patterns, whereas sex had minimal effects, suggesting that individual brain organization plays an important role in shaping functional lateralization rather than solely motor factors. Finally, these findings emphasize the need to account for task conditions and demographic factors when using EEG asymmetry as a clinical biomarker to avoid misinterpretation in diagnostic and therapeutic contexts. These findings will be discussed in detail in the following sections, focusing on (1) differences between resting-state and task-related asymmetry, (2) the role of motor execution in shaping asymmetry, (3) the influence of demographic factors such as age and handedness, and (4) the implications for clinical research and applications.

### 4.1 Differences between resting-state and task-related asymmetry

First, this study found that task-related EEG asymmetries showed dynamic changes from resting-state, but the patterns were not uniform across tasks or frequency bands. In particular, left-sided asymmetries in the alpha band were observed during resting-state and after stimulus presentation in the PVT, suggesting that factors beyond simple motor execution may contribute. While one possibility is interaction with perceptual or cognitive processes, the PVT has minimal visual demands, so these mechanisms remain speculative. Interestingly, no significant changes in asymmetry were found in the bimanual tasks (Simon and Stroop), highlighting that task-related asymmetry may depend on the motor configuration and cannot be generalized across all tasks. The most substantial changes occurred when comparing resting-state or pre-stimulus asymmetries to post-stimulus asymmetries, supporting the idea that functional lateralization is dynamic and modulated by cognitive and motor demands. These findings align with prior research examining motor-related lateralization. For example, Meng et al. (2008) investigated left–right differences in event-related potentials associated with right-hand finger tapping. Their study analyzed neural activity across different processing phases: early visual processing, pre-execution, execution, and post-execution. They reported that event-related potentials at left-hemispheric electrodes (e.g., F3, C3, P3, and O1) were significantly more pronounced than those at right-hemispheric electrodes (e.g., F4, C4, P4, and O2) during the execution phase. Additionally, while no significant left–right differences were observed in early visual processing or other phases, the pre- and post-execution phases did show some left dominance, particularly at the C3. These findings by Meng et al. (2008) suggest that unilateral motor execution engages the left hemisphere not only during movement but also in preparatory and post-movement processing, supporting the notion that functional lateralization is task-dependent and dynamically modulated by the task processing phase (Meng et al., 2008). However, it is worth noting that the absence of medium effect sizes in the Stroop task may partly reflect differences in EEG systems, such as the use of 32-channel versus 64-channel electrode setups, which could introduce additional noise and reduce sensitivity to certain effects. Moreover, task-specific cognitive demands and inter-individual variability likely contribute to the observed pattern. Thus, the Stroop results should be interpreted cautiously, taking into account both methodological and task-related influences.

The findings of left-sided asymmetries in our study might reflect a combination of intrinsic neural organization, motor execution, and possibly perceptual processes, although our data do not allow firm conclusions regarding the relative contribution of each factor. The observed left-sided asymmetry may also be explained by the asymmetric functional contributions of frontoparietal circuits linked by the superior longitudinal fasciculus, which connects regions involved in motor execution, spatial attention, and perceptual decision-making and has been strongly implicated in neglect-related deficits (Lunven et al., 2015; Janelle et al., 2022). Recent human intracerebral recordings indicate that the right hemisphere is crucial for attention-based prioritization of information, whereas the left hemisphere plays a dominant role in perceptual decision-making and model building (Bartolomeo et al., 2024). These hemispheric asymmetries align with clinical evidence from neglect patients and suggest that the left-lateralized effects observed in this study may reflect an interaction between motor execution and perceptual processes (Doricchi and Tomaiuolo, 2003; Thiebaut de Schotten et al., 2014; Lunven et al., 2015). Specifically, damage to the superior longitudinal fasciculus has been strongly associated with spatial neglect, highlighting the importance of frontoparietal disconnection in shaping attentional and sensorimotor asymmetries. Thiebaut de Schotten et al. (2014) demonstrated that lesions in the second branch of the superior longitudinal fasciculus (SLF II) are the strongest predictor of chronic left spatial neglect (Thiebaut de Schotten et al., 2014), while Lunven et al. (2015) showed that persistent neglect is linked to both frontoparietal and interhemispheric disconnection, particularly involving the splenium of the corpus callosum (Lunven et al., 2015). These findings suggest that the left hemisphere may not fully compensate for right-hemisphere damage, leading to enduring perceptual and motor asymmetries. This further reinforces the idea that functional lateralization in EEG is shaped not only by motor demands, but also by underlying structural and cognitive asymmetries in the brain.

### 4.2 The role of motor execution in shaping asymmetry

Second, the observed shifts in asymmetry predominantly occurred post-stimulus, suggesting that motor execution contributes under the PVT conditions, though preparatory or cognitive factors may also play a role. While some theories suggest that asymmetries in the alpha band reflect preparatory processes (Meng et al., 2008; Deiber et al., 2012), the observed shifts in asymmetry occurred predominantly after stimulus presentation, suggesting that the act of responding, rather than mere preparedness to respond, contributes to the observed changes. The frequency-specific effects further support this interpretation. The fact that theta, alpha, and beta bands were influenced by timepoint suggests distinct functional roles. Alpha activity has been demonstrated to facilitate inhibitory processes (Knyazev, 2007), and right-hemispheric fronto-parietal networks have been strongly implicated in spatial attention and attentional prioritization, forming a key component of right-lateralized attention systems (Corbetta and Shulman, 2002). Frontal midline theta activity has been linked to cognitive control, performance monitoring, and working memory demands, typically involving medial prefrontal regions (Cavanagh and Frank, 2014). The beta band is hypothesized to signal the ‘status quo’ for movement and cognition (Engel and Fries, 2010), and beta-band activity in sensorimotor regions typically decreases during movement and increases again after movement ends (Neuper and Pfurtscheller, 2001). This post-movement increase has been interpreted to reflect temporary suppression of motor activity and a reset of the motor system after action execution (Neuper and Pfurtscheller, 2001). The highest frequency band, i.e., the gamma band, has been shown to increase in response to any form of change or demand for attention (Laufs et al., 2006). Stronger interhemispheric coupling observed in the alpha and beta bands in frontal regions after stimulus presentation might suggest increased neural integration during cognitive and motor tasks. In contrast, weaker connectivity in central regions, particularly in the theta band, might reflect more localized task-specific processing rather than broad interhemispheric coordination.

### 4.3 Influence of demographic factors such as age and handedness

Third, demographic factors such as age and handedness influenced EEG asymmetry across different tasks and frequency bands, while sex had a minimal effect. Moreover, the interaction between age and electrode pair was more pronounced in non-right-handed individuals, particularly in the alpha and beta frequency bands, potentially indicating that electrode location had a stronger impact on EEG asymmetry in this group. Additionally, non-right-handed individuals exhibited greater variation in asymmetry shifts, with asymmetries observed not only in the alpha band, as seen in right-handed individuals, but also in the theta and delta bands at rest. Right-handed individuals, on the other hand, displayed a more focused pattern, with timepoint effects primarily influencing alpha activity across tasks. In contrast, non-right-handed individuals showed a broader range of timing effects, with theta, alpha, and beta frequencies all significantly influenced by timepoint. Notably, the group of non-right-handers was substantially smaller than the group of right-handers, which could have affected the different results. Also, the blocks of the Stroop task were not separated, which could have led to the weak effect sizes found. Nevertheless, one possible explanation for these findings could be structural and functional differences in brain organization between right- and non-right-handed individuals. A compelling explanation would be that differences in callosal connectivity between right- and non-right-handers lead to differences in interhemispheric integration. However, recent large-scale studies and meta-analyses have not confirmed differences between handedness groups (Westerhausen and Papadatou-Pastou, 2022; Raaf and Westerhausen, 2023). Thus, differences in callosal connectivity do not explain why non-right-handed individuals showed more dynamic shifts in EEG asymmetry across tasks and frequency bands, while right-handed individuals exhibited more stable lateralization patterns. On the other hand, research suggests that stronger right-handedness is associated with more pronounced rightward alpha asymmetry or increased left-hemisphere activity (Ocklenburg et al., 2019). Furthermore, the fact that non-right-handed individuals showed asymmetry shifts in theta, alpha, and beta across all time points may suggest that their neural organization is more flexible and dynamically adapts across task demands. This supports the idea that greater interhemispheric communication in non-right-handed individuals may allow for more distributed processing over time rather than fixed lateralization patterns. Further supporting this idea, Gotts et al. (2013) demonstrated that hemispheric lateralization follows distinct functional principles, with the left hemisphere showing a preference for intra-hemispheric processing, particularly in language and fine motor coordination, while the right hemisphere engages in a more inter-hemispheric, integrative processing mode, particularly for visuospatial and attentional functions (Gotts et al., 2013). These findings suggest that individual differences in lateralization may contribute to variations in cognitive efficiency, particularly in tasks relying on motor execution and attentional control. However, this relationship cannot be conclusively disentangled with the present data. At the same time, Learmonth et al. (2017) provide evidence that hemispheric lateralization is not static but changes across the lifespan, particularly in the context of spatial attention. Their study found that while younger adults exhibit a leftward visuospatial bias (i.e., pseudoneglect), driven by right-hemispheric dominance for spatial attention, older adults show a rightward shift, suggesting that the right hemisphere’s role in spatial attention weakens with age. Corresponding EEG evidence supports this, showing a reduction in right-hemispheric specialization in older adults, leading to more bilateral, and possibly less efficient, processing (Learmonth et al., 2017). These findings indicate that age-related reductions in asymmetry may reflect compensatory mechanisms or early neurodegenerative changes rather than a simple loss of function, in line with the neural dedifferentiation hypothesis of aging (e.g., Goh, 2011; Koen and Rugg, 2019).

By combining these perspectives, it becomes clear that functional lateralization is shaped by both inherent neural organization and age-related changes. While the left hemisphere’s specialization for fine motor and language-related processing may remain relatively stable across individuals, the right hemisphere’s role in spatial and attentional processing may appear more flexible, diminishing with age. This would have important implications for interpreting EEG asymmetry, as shifts in lateralization may reflect individual differences in brain organization, compensatory neural mechanisms, or pathological changes depending on the context. Failing to account for task-specific effects, demographic factors, and timepoint variations could lead to misinterpretations when using EEG asymmetry as a diagnostic or cognitive marker.

### 4.4 Implications for clinical research and applications

Fourth, the observed patterns of EEG asymmetry suggest potential applications as a clinical and cognitive marker. The fact that EEG asymmetry varies with task type, handedness, and age suggests that clinicians must carefully consider these factors when interpreting EEG data. EEG asymmetry is often used as a biomarker for neuropsychiatric disorders (Thibodeau et al., 2006; Nusslock et al., 2018; Smith et al., 2018). However, if asymmetry patterns systematically change with age and handedness, failing to consider these factors could lead to misinterpretation. For example, an observed asymmetry in an older adult or a non-right-handed individual may not necessarily indicate pathology but could instead reflect normative variations in brain organization. In retrospect, a more fine-grained examination of how handedness relates to hemisphere-specific activity would have further strengthened the interpretation of these effects. However, the present study was not designed or powered to address this question in detail beyond the comparison between right- and non-right-handed groups.

The task-specific patterns observed, including the absence of significant effects in the Stroop task, emphasize the need for descriptive evaluation of EEG asymmetry across multiple conditions. Mapping these variations can help characterize how asymmetry emerges under different cognitive and motor demands and may inform applications in neurocognitive assessment or brain-computer interface development (Krogmeier et al., 2022).

## 5 Limitations and strengths

Analysis focused on only three cognitive tasks, which might not capture the full spectrum of cognitive and emotional processes influencing EEG asymmetry. While minimal effects of age and sex were observed, individual differences could still play a role in more specific contexts. The study also does not delve into the underlying neural mechanisms driving the observed lateralization shifts, leaving room for further exploration. Given the limited spatial resolution of EEG, we cannot precisely attribute the observed asymmetry effects to distinct motor and/or cognitive sources. Asymmetry was analyzed at the sensor level, which means that volume conduction and reference effects may have influenced the observed patterns and should be considered when interpreting the results. Moreover, two different EEG systems were used across tasks, which may have influenced asymmetry measures. Although previous work (Metzen et al., 2022) suggests relative asymmetry is generally reliable across setups, this remains a methodological limitation for cross-task comparisons. A further limitation of the present study is that different band-pass filters were applied to resting-state and task-related EEG data. This may have influenced the magnitude of condition differences, as very low-frequency activity (<1 Hz) was only included in the task condition. While asymmetry measures are based on relative differences and are therefore likely robust to such effects, the impact on task-rest comparisons cannot be fully ruled out and may have resulted in more conservative estimates. As the sample mainly consists of healthy individuals from the Dortmund Vital Study, the generalizability of the results might be limited.

Despite these limitations, the study’s strengths include its large sample size of 610 participants, spanning a broad adult age range (20–70 years), which enhances the reliability of the results. By incorporating both resting-state and task-based measures across multiple frequency bands, the study effectively explores how motor and cognitive demands influence EEG asymmetry. This systematic cross-task comparison provides valuable insights into individual differences in lateralization, particularly for non-right-handed individuals, and sheds light on the dynamic nature of interhemispheric coupling in frontal and central regions. Furthermore, the dataset has potential as a normative reference. These contributions are important for refining the interpretation of EEG biomarkers in both research and clinical settings.

## 6 Clinical implications

A key takeaway for clinical researchers based on the findings of the present study is that EEG asymmetry, particularly in the alpha, beta, and theta frequency bands, appears to be influenced by both task demands and motor execution in addition to cognitive processing. This suggests that interpreting EEG asymmetry in clinical settings requires careful consideration of task-related motor activity to avoid misattributing lateralization effects to purely cognitive or emotional processes. Furthermore, the minimal effects of sex on EEG asymmetry and the stronger influence of age and handedness highlight the importance of accounting for individual differences when using EEG as a diagnostic or monitoring tool. These findings reinforce the need for standardized protocols that differentiate between resting-state and task-related asymmetry to improve the reliability of EEG-based biomarkers for neurocognitive and psychiatric disorders. With its exceptionally large sample of EEG participants, this study offers a solid foundation for the development of such standards and may serve as a reference for future clinical and non-clinical studies.

## 7 Conclusion

This study aimed to enhance the interpretive power of EEG in clinical settings by clarifying whether EEG frequency band asymmetries reflect cognitive processes or are influenced by motor processes. Results demonstrate that task-related EEG asymmetries differ from resting-state asymmetries, with motor execution and cognitive processes influencing lateralization patterns. This highlights the need for additional consideration of motor activity when interpreting EEG asymmetry in both research and clinical applications. Moreover, the study found that demographic factors such as age and handedness influence EEG asymmetry, while sex has minimal impact. These results emphasize the importance of accounting for individual differences when using EEG as a diagnostic or monitoring tool. By distinguishing between task-related and intrinsic asymmetry, these insights could contribute to the development of more precise biomarkers, ultimately improving the clinical utility of EEG for diagnosing and monitoring neurocognitive and psychiatric disorders.

## Data Availability

The data analyzed in this study is subject to the following licenses/restrictions: Data will be made available upon reasonable request to the corresponding author. The study was registered at ClinicalTrials.gov: NCT05155397. Requests to access these datasets should be directed to annakarina.mundorf@medicalschool-hamburg.de.
