# Supplementary material for: Effects of motor activity versus cognitive demand on asymmetries in EEG frequency bands and their relation to handedness
Source: Front Hum Neurosci. 2026 Jul 8;20:1796350. doi: 10.3389/fnhum.2026.1796350 (PMC13388754; doi:10.3389/fnhum.2026.1796350)
Supplement: Supplementary file 1 [file Data_Sheet_1.ZIP › Supplements/Supplementary Material 3_Figures.docx]

**Supplementary Material 3:**

**Effects of motor activity versus cognitive demand on asymmetries in EEG frequency bands and their relation to handedness (Mundorf et al., 2026)**

**Suppl. Figure 1.** Interaction effect sizes (*η_p_^2^*) derived from the ANOVAs for each electrode pair and timepoint during resting-state and in the Psychomotor Vigilance Task (PVT; pre- and post-stimulus) in right-handed individuals. The color scale reflects effect size magnitude, ranging from dark blue (smaller effect sizes) to light blue and orange, with red indicating the largest effect sizes.

**Suppl. Figure 2.** Interaction effect sizes (*η_p_^2^*) derived from the ANOVAs for each electrode pair and timepoint during resting-state and in the Simon Task (pre- and post-stimulus) in right-handed individuals. The color scale reflects effect size magnitude, ranging from dark blue (smaller effect sizes) to light blue and orange, with red indicating the largest effect sizes.

**Suppl. Figure 3.** Interaction effect sizes (*η_p_^2^*) derived from the ANOVAs for each electrode pair and timepoint during resting-state and in the Stroop Task (pre- and post-stimulus) in right-handed individuals. The color scale reflects effect size magnitude, ranging from dark blue (smaller effect sizes) to light blue and orange, with red indicating the largest effect sizes.

**Suppl. Figure 4.** Interaction effect sizes (*η_p_^2^*) derived from the ANOVAs for each electrode pair and timepoint during resting-state and in the PVT (pre- and post-stimulus) in non-right-handed individuals. The color scale reflects effect size magnitude, ranging from dark blue (smaller effect sizes) to light blue and orange, with red indicating the largest effect sizes.

**Suppl. Figure 5.** Interaction effect sizes (*η_p_^2^*) derived from the ANOVAs for each electrode pair and timepoint during resting-state and in the Simon Task (pre- and post-stimulus) in non-right-handed individuals. The color scale reflects effect size magnitude, ranging from dark blue (smaller effect sizes) to light blue and orange, with red indicating the largest effect sizes.

**Suppl. Figure 6.** Interaction effect sizes (*η_p_^2^*) derived from the ANOVAs for each electrode pair and timepoint during resting-state and in the Stroop Task (pre- and post-stimulus) in non-right-handed individuals. The color scale reflects effect size magnitude, ranging from dark blue (smaller effect sizes) to light blue and orange, with red indicating the largest effect sizes.

**Suppl. Figure 7.** Asymmetry Index (AI) as a function of age (in years) in the alpha frequency band during resting-state and in the Psychomotor Vigilance Task (PVT; pre- and post-stimulus) in right-handed individuals. Positive scores reflect higher right-hemispheric power and negative scores reflect higher left-hemispheric power. The color coding indicates different electrode pairs, with each pair assigned a distinct color gradient for visual separation: C3/C4 (red), CP3/CP4 (orange), F3/F4 (green-orange), F7/F8 (light green), FC3/FC4 (green), Fp1/Fp2 (darker green), O1/O2 (light blue), P3/P4 (dark blue), P7/P8 (purple), PO3/PO4 (light purple), and T7/T8 (pink).

**Suppl. Figure 8.** Asymmetry Index (AI) as a function of age (in years) in the beta frequency band during resting-state and in the Psychomotor Vigilance Task (PVT; pre- and post-stimulus) in right-handed individuals. Positive scores reflect higher right-hemispheric power and negative scores reflect higher left-hemispheric power. The color coding indicates different electrode pairs, with each pair assigned a distinct color gradient for visual separation: C3/C4 (red), CP3/CP4 (orange), F3/F4 (green-orange), F7/F8 (light green), FC3/FC4 (green), Fp1/Fp2 (darker green), O1/O2 (light blue), P3/P4 (dark blue), P7/P8 (purple), PO3/PO4 (light purple), and T7/T8 (pink).

**Suppl. Figure 9**. Asymmetry Index (AI) as a function of age (in years) in the delta frequency band during resting-state and in the Psychomotor Vigilance Task (PVT; pre- and post-stimulus) in right-handed individuals. Positive scores reflect higher right-hemispheric power and negative scores reflect higher left-hemispheric power. The color coding indicates different electrode pairs, with each pair assigned a distinct color gradient for visual separation: C3/C4 (red), CP3/CP4 (orange), F3/F4 (green-orange), F7/F8 (light green), FC3/FC4 (green), Fp1/Fp2 (darker green), O1/O2 (light blue), P3/P4 (dark blue), P7/P8 (purple), PO3/PO4 (light purple), and T7/T8 (pink).

**Suppl. Figure 10.** Asymmetry Index (AI) as a function of age (in years) in the theta frequency band during resting-state and in the Psychomotor Vigilance Task (PVT; pre- and post-stimulus) in right-handed individuals. Positive scores reflect higher right-hemispheric power and negative scores reflect higher left-hemispheric power. The color coding indicates different electrode pairs, with each pair assigned a distinct color gradient for visual separation: C3/C4 (red), CP3/CP4 (orange), F3/F4 (green-orange), F7/F8 (light green), FC3/FC4 (green), Fp1/Fp2 (darker green), O1/O2 (light blue), P3/P4 (dark blue), P7/P8 (purple), PO3/PO4 (light purple), and T7/T8 (pink).

**Suppl. Figure 11.** Asymmetry Index (AI) as a function of age (in years) in the alpha frequency band during resting-state and in the Simon Task (pre- and post-stimulus) in right-handed individuals. Positive scores reflect higher right-hemispheric power and negative scores reflect higher left-hemispheric power. The color coding indicates different electrode pairs, with each pair assigned a distinct color gradient for visual separation: C3/C4 (red), CP3/CP4 (orange), F3/F4 (green-orange), F7/F8 (light green), FC3/FC4 (green), Fp1/Fp2 (darker green), O1/O2 (light blue), P3/P4 (dark blue), P7/P8 (purple), PO3/PO4 (light purple), and T7/T8 (pink).

**Suppl. Figure 12.** Asymmetry Index (AI) as a function of age (in years) in the beta frequency band during resting-state and in the Simon Task (pre- and post-stimulus) in right-handed individuals. Positive scores reflect higher right-hemispheric power and negative scores reflect higher left-hemispheric power. The color coding indicates different electrode pairs, with each pair assigned a distinct color gradient for visual separation: C3/C4 (red), CP3/CP4 (orange), F3/F4 (green-orange), F7/F8 (light green), FC3/FC4 (green), Fp1/Fp2 (darker green), O1/O2 (light blue), P3/P4 (dark blue), P7/P8 (purple), PO3/PO4 (light purple), and T7/T8 (pink).

**Suppl. Figure 13**. Asymmetry Index (AI) as a function of age (in years) in the delta frequency band during resting-state and in the Simon Task (pre- and post-stimulus) in right-handed individuals. Positive scores reflect higher right-hemispheric power and negative scores reflect higher left-hemispheric power. The color coding indicates different electrode pairs, with each pair assigned a distinct color gradient for visual separation: C3/C4 (red), CP3/CP4 (orange), F3/F4 (green-orange), F7/F8 (light green), FC3/FC4 (green), Fp1/Fp2 (darker green), O1/O2 (light blue), P3/P4 (dark blue), P7/P8 (purple), PO3/PO4 (light purple), and T7/T8 (pink).

**Suppl. Figure 14.** Asymmetry Index (AI) as a function of age (in years) in the theta frequency band during resting-state and in the Simon Task (pre- and post-stimulus) in right-handed individuals. Positive scores reflect higher right-hemispheric power and negative scores reflect higher left-hemispheric power. The color coding indicates different electrode pairs, with each pair assigned a distinct color gradient for visual separation: C3/C4 (red), CP3/CP4 (orange), F3/F4 (green-orange), F7/F8 (light green), FC3/FC4 (green), Fp1/Fp2 (darker green), O1/O2 (light blue), P3/P4 (dark blue), P7/P8 (purple), PO3/PO4 (light purple), and T7/T8 (pink).

**Suppl. Figure 15.** Asymmetry Index (AI) as a function of age (in years) in the alpha frequency band during resting-state and in the Stroop Task (pre- and post-stimulus) in right-handed individuals. Positive scores reflect higher right-hemispheric power and negative scores reflect higher left-hemispheric power. The color coding indicates different electrode pairs, with each pair assigned a distinct color gradient for visual separation: C3/C4 (red), CP3/CP4 (orange), F3/F4 (green-orange), F7/F8 (light green), FC3/FC4 (green), Fp1/Fp2 (darker green), O1/O2 (light blue), P3/P4 (dark blue), P7/P8 (purple), PO3/PO4 (light purple), and T7/T8 (pink).

**Suppl. Figure 16.** Asymmetry Index (AI) as a function of age (in years) in the beta frequency band during resting-state and in the Stroop Task (pre- and post-stimulus) in right-handed individuals. Positive scores reflect higher right-hemispheric power and negative scores reflect higher left-hemispheric power. The color coding indicates different electrode pairs, with each pair assigned a distinct color gradient for visual separation: C3/C4 (red), CP3/CP4 (orange), F3/F4 (green-orange), F7/F8 (light green), FC3/FC4 (green), Fp1/Fp2 (darker green), O1/O2 (light blue), P3/P4 (dark blue), P7/P8 (purple), PO3/PO4 (light purple), and T7/T8 (pink).

**Suppl. Figure 17**. Asymmetry Index (AI) as a function of age (in years) in the delta frequency band during resting-state and in the Stroop Task (pre- and post-stimulus) in right-handed individuals. Positive scores reflect higher right-hemispheric power and negative scores reflect higher left-hemispheric power. The color coding indicates different electrode pairs, with each pair assigned a distinct color gradient for visual separation: C3/C4 (red), CP3/CP4 (orange), F3/F4 (green-orange), F7/F8 (light green), FC3/FC4 (green), Fp1/Fp2 (darker green), O1/O2 (light blue), P3/P4 (dark blue), P7/P8 (purple), PO3/PO4 (light purple), and T7/T8 (pink).

**Suppl. Figure 18.** Asymmetry Index (AI) as a function of age (in years) in the theta frequency band during resting-state and in the Stroop Task (pre- and post-stimulus) in right-handed individuals. Positive scores reflect higher right-hemispheric power and negative scores reflect higher left-hemispheric power. The color coding indicates different electrode pairs, with each pair assigned a distinct color gradient for visual separation: C3/C4 (red), CP3/CP4 (orange), F3/F4 (green-orange), F7/F8 (light green), FC3/FC4 (green), Fp1/Fp2 (darker green), O1/O2 (light blue), P3/P4 (dark blue), P7/P8 (purple), PO3/PO4 (light purple), and T7/T8 (pink).

**Suppl. Figure 19.** Asymmetry Index (AI) as a function of age (in years) in the alpha frequency band during resting state and in the Psychomotor Vigilance Task (PVT; pre- and post-stimulus) in non-right-handed individuals. Positive scores reflect higher right-hemispheric power, and negative scores reflect higher left-hemispheric power. The color coding indicates different electrode pairs, with each pair assigned a distinct color gradient for visual separation: C3/C4 (red), CP3/CP4 (orange), F3/F4 (green-orange), F7/F8 (light green), FC3/FC4 (green), Fp1/Fp2 (darker green), O1/O2 (light blue), P3/P4 (dark blue), P7/P8 (purple), PO3/PO4 (light purple), and T7/T8 (pink).

**Suppl. Figure 20.** Asymmetry Index (AI) as a function of age (in years) in the beta frequency band during resting state and in the Psychomotor Vigilance Task (PVT; pre- and post-stimulus) in non-right-handed individuals. Positive scores reflect higher right-hemispheric power and negative scores reflect higher left-hemispheric power. The color coding indicates different electrode pairs, with each pair assigned a distinct color gradient for visual separation: C3/C4 (red), CP3/CP4 (orange), F3/F4 (green-orange), F7/F8 (light green), FC3/FC4 (green), Fp1/Fp2 (darker green), O1/O2 (light blue), P3/P4 (dark blue), P7/P8 (purple), PO3/PO4 (light purple), and T7/T8 (pink).

**Suppl. Figure 21.** Asymmetry Index (AI) as a function of age (in years) in the delta frequency band during resting state and in the Psychomotor Vigilance Task (PVT; pre- and post-stimulus) in non-right-handed individuals. Positive scores reflect higher right-hemispheric power and negative scores reflect higher left-hemispheric power. The color coding indicates different electrode pairs, with each pair assigned a distinct color gradient for visual separation: C3/C4 (red), CP3/CP4 (orange), F3/F4 (green-orange), F7/F8 (light green), FC3/FC4 (green), Fp1/Fp2 (darker green), O1/O2 (light blue), P3/P4 (dark blue), P7/P8 (purple), PO3/PO4 (light purple), and T7/T8 (pink).

**Suppl. Figure 22.** Asymmetry Index (AI) as a function of age (in years) in the theta frequency band during resting state and in the Psychomotor Vigilance Task (PVT; pre- and post-stimulus) in non-right-handed individuals. Positive scores reflect higher right-hemispheric power and negative scores reflect higher left-hemispheric power. The color coding indicates different electrode pairs, with each pair assigned a distinct color gradient for visual separation: C3/C4 (red), CP3/CP4 (orange), F3/F4 (green-orange), F7/F8 (light green), FC3/FC4 (green), Fp1/Fp2 (darker green), O1/O2 (light blue), P3/P4 (dark blue), P7/P8 (purple), PO3/PO4 (light purple), and T7/T8 (pink).

**Suppl. Figure 23.** Asymmetry Index (AI) as a function of age (in years) in the alpha frequency band during resting-state and in the Simon Task (pre- and post-stimulus) in non-right-handed individuals. Positive scores reflect higher right-hemispheric power and negative scores reflect higher left-hemispheric power. The color coding indicates different electrode pairs, with each pair assigned a distinct color gradient for visual separation: C3/C4 (red), CP3/CP4 (orange), F3/F4 (green-orange), F7/F8 (light green), FC3/FC4 (green), Fp1/Fp2 (darker green), O1/O2 (light blue), P3/P4 (dark blue), P7/P8 (purple), PO3/PO4 (light purple), and T7/T8 (pink).

**Suppl. Figure 24.** Asymmetry Index (AI) as a function of age (in years) in the beta frequency band during resting state and in the Simon Task (pre- and post-stimulus) in non-right-handed individuals. Positive scores reflect higher right-hemispheric power and negative scores reflect higher left-hemispheric power. The color coding indicates different electrode pairs, with each pair assigned a distinct color gradient for visual separation: C3/C4 (red), CP3/CP4 (orange), F3/F4 (green-orange), F7/F8 (light green), FC3/FC4 (green), Fp1/Fp2 (darker green), O1/O2 (light blue), P3/P4 (dark blue), P7/P8 (purple), PO3/PO4 (light purple), and T7/T8 (pink).

**Suppl. Figure 25.** Asymmetry Index (AI) as a function of age (in years) in the delta frequency band during resting-state and in the Simon Task (pre- and post-stimulus) in non-right-handed individuals. Positive scores reflect higher right-hemispheric power and negative scores reflect higher left-hemispheric power. The color coding indicates different electrode pairs, with each pair assigned a distinct color gradient for visual separation: C3/C4 (red), CP3/CP4 (orange), F3/F4 (green-orange), F7/F8 (light green), FC3/FC4 (green), Fp1/Fp2 (darker green), O1/O2 (light blue), P3/P4 (dark blue), P7/P8 (purple), PO3/PO4 (light purple), and T7/T8 (pink).

**Suppl. Figure 26.** Asymmetry Index (AI) as a function of age (in years) in the theta frequency band during resting-state and in the Simon Task (pre- and post-stimulus) in non-right-handed individuals. Positive scores reflect higher right-hemispheric power and negative scores reflect higher left-hemispheric power. The color coding indicates different electrode pairs, with each pair assigned a distinct color gradient for visual separation: C3/C4 (red), CP3/CP4 (orange), F3/F4 (green-orange), F7/F8 (light green), FC3/FC4 (green), Fp1/Fp2 (darker green), O1/O2 (light blue), P3/P4 (dark blue), P7/P8 (purple), PO3/PO4 (light purple), and T7/T8 (pink).

**Suppl. Figure 27.** Asymmetry Index (AI) as a function of age (in years) in the alpha frequency band during resting-state and in the Stroop Task (pre- and post-stimulus) in non-right-handed individuals. Positive scores reflect higher right-hemispheric power and negative scores reflect higher left-hemispheric power. The color coding indicates different electrode pairs, with each pair assigned a distinct color gradient for visual separation: C3/C4 (red), CP3/CP4 (orange), F3/F4 (green-orange), F7/F8 (light green), FC3/FC4 (green), Fp1/Fp2 (darker green), O1/O2 (light blue), P3/P4 (dark blue), P7/P8 (purple), PO3/PO4 (light purple), and T7/T8 (pink).

**Suppl. Figure 28.** Asymmetry Index (AI) as a function of age (in years) in the beta frequency band during resting-state and in the Stroop Task (pre- and post-stimulus) in non-right-handed individuals. Positive scores reflect higher right-hemispheric power and negative scores reflect higher left-hemispheric power. The color coding indicates different electrode pairs, with each pair assigned a distinct color gradient for visual separation: C3/C4 (red), CP3/CP4 (orange), F3/F4 (green-orange), F7/F8 (light green), FC3/FC4 (green), Fp1/Fp2 (darker green), O1/O2 (light blue), P3/P4 (dark blue), P7/P8 (purple), PO3/PO4 (light purple), and T7/T8 (pink).

**Suppl. Figure 29.** Asymmetry Index (AI) as a function of age (in years) in the delta frequency band during resting-state and in the Stroop Task (pre- and post-stimulus) in non-right-handed individuals. Positive scores reflect higher right-hemispheric power and negative scores reflect higher left-hemispheric power. The color coding indicates different electrode pairs, with each pair assigned a distinct color gradient for visual separation: C3/C4 (red), CP3/CP4 (orange), F3/F4 (green-orange), F7/F8 (light green), FC3/FC4 (green), Fp1/Fp2 (darker green), O1/O2 (light blue), P3/P4 (dark blue), P7/P8 (purple), PO3/PO4 (light purple), and T7/T8 (pink).

**Suppl. Figure 30.** Asymmetry Index (AI) as a function of age (in years) in the theta frequency band during resting-state and in the Stroop Task (pre- and post-stimulus) in non-right-handed individuals. Positive scores reflect higher right-hemispheric power and negative scores reflect higher left-hemispheric power. The color coding indicates different electrode pairs, with each pair assigned a distinct color gradient for visual separation: C3/C4 (red), CP3/CP4 (orange), F3/F4 (green-orange), F7/F8 (light green), FC3/FC4 (green), Fp1/Fp2 (darker green), O1/O2 (light blue), P3/P4 (dark blue), P7/P8 (purple), PO3/PO4 (light purple), and T7/T8 (pink).

**Suppl. Figure 31**. Mean Asymmetry Index (AI) and SD for each electrode pair in the PVT task pre- and post-stimuli and resting-state for the frequency bands (A) alpha, (B) beta, (C) delta and (D) theta. Positive scores reflect higher right-hemispheric power, and negative scores reflect higher left-hemispheric power. All asymmetries with medium effect sizes (d = -0.52 to -0.74) indicated left-sided asymmetries and were observed in the alpha frequency band during resting-state in parietal regions (electrode pairs: P3/P4, P7/P8) and in the PVT post-stimulus in parietal regions (electrode pairs: CP3/CP4, P3/P4). Effect sizes and statistical results for all reported asymmetries are presented in Supplementary Material 7. The color coding indicates task condition: blue (pre-stimulus), red (post-stimulus), and yellow (resting-state).

**t-tests against zero results significantly differed from zero (-0.5 ≤ d < -0.8).*

**Suppl. Figure 32**. Mean Asymmetry Index (AI) and SD for each electrode pair in the Simon task pre- and post-stimuli and resting-state for the frequency bands (A) alpha, (B) beta, (C) delta and (D) theta. Positive scores reflect higher right-hemispheric power, and negative scores reflect higher left-hemispheric power. All asymmetries with medium effect sizes (d = -0.52 to -0.74) indicated left-sided asymmetries and were observed in the alpha frequency band during resting-state in parietal regions (electrode pairs: P3/P4, P7/P8). Effect sizes and statistical results for all reported asymmetries are presented in Supplementary Material 7. The color coding indicates task condition: blue (pre-stimulus), red (post-stimulus), and yellow (resting-state).

** t-tests against zero results significantly differed from zero (-0.5 ≤ d < -0.8)*

**Suppl. Figure 33**. Mean Asymmetry Index (AI) and SD for each electrode pair in the Stroop task pre- and post-stimuli and resting-state for the frequency bands (A) alpha, (B) beta, (C) delta and (D) theta. Positive scores reflect higher right-hemispheric power, and negative scores reflect higher left-hemispheric power. All asymmetries with medium effect sizes (d = -0.52 to -0.74) indicated left-sided asymmetries and were observed in the alpha frequency band during resting-state in parietal regions (electrode pairs: P3/P4, P7/P8). Effect sizes and statistical results for all reported asymmetries are presented in Supplementary Material 7. The color coding indicates task condition: blue (pre-stimulus), red (post-stimulus), and yellow (resting-state).

**t-tests against zero results significantly differed from zero (-0.5 ≤ d < -0.8)*

**Suppl. Figure 34.** Mean Asymmetry Index (AI) and SD for each electrode pair in the PVT task pre- and post-stimuli and resting-state for the frequency bands (A) alpha, (B) beta, (C) delta and (D) theta in non-right-handers. Positive scores reflect higher right-hemispheric power, and negative scores reflect higher left-hemispheric power. All asymmetries with medium effect sizes indicated left-sided asymmetries and were observed during resting-state in parietal regions in the alpha (electrode pairs: P3/P4, P7/P8; d = -0.63 to -0.66), theta (electrode pair: P3/P4; d = -0.51), and delta frequency bands (electrode pair: P3/P4; d = -0.62). During the post-stimulus PVT condition, medium effect sizes indicating left-sided asymmetries were observed in the alpha frequency band in parietal regions (electrode pairs: CP3/CP4, P3/P4, PO3/PO4; d = -0.52 to -0.76). Effect sizes and statistical results for all reported asymmetries are presented in Supplementary Material 7. The color coding indicates task condition: blue (pre-stimulus), red (post-stimulus), and yellow (resting-state).

**t-tests against zero results significantly differed from zero (-0.5 ≤ d < -0.8)*

**Suppl. Figure 35.** Mean Asymmetry Index (AI) and SD for each electrode pair in the Simon task pre- and post-stimuli and resting-state for the frequency bands (A) alpha, (B) beta, (C) delta and (D) theta in non-right-handers. Positive scores reflect higher right-hemispheric power, and negative scores reflect higher left-hemispheric power. All asymmetries with medium effect sizes indicated left-sided asymmetries and were observed during resting-state in parietal regions in the alpha (electrode pairs: P3/P4, P7/P8; d = -0.63 to -0.66), theta (electrode pair: P3/P4; d = -0.51), and delta frequency bands (electrode pair: P3/P4; d = -0.62). Effect sizes and statistical results for all reported asymmetries are presented in Supplementary Material 7. The color coding indicates task condition: blue (pre-stimulus), red (post-stimulus), and yellow (resting-state).

**t-tests against zero results significantly differed from zero (-0.5 ≤ d < -0.8)*

**Suppl. Figure 36**. Mean Asymmetry Index (AI) and SD for each electrode pair in the Stroop task pre- and post-stimuli and resting-state for the frequency bands (A) alpha, (B) beta, (C) delta, and (D) theta for non-right-handers. Positive scores reflect higher right-hemispheric power, and negative scores reflect higher left-hemispheric power. All asymmetries with medium effect sizes indicated left-sided asymmetries and were observed during resting-state in parietal regions in the alpha (electrode pairs: P3/P4, P7/P8; d = -0.63 to -0.66), theta (electrode pair: P3/P4; d = -0.51), and delta frequency bands (electrode pair: P3/P4; d = -0.62). The color coding indicates task condition: blue (pre-stimulus), red (post-stimulus), and yellow (resting-state).

**t-tests against zero results significantly differed from zero (-0.5 ≤ d < -0.8)*
